# Ginger on Human Health: A Comprehensive Systematic Review of 109 Randomized Controlled Trials

**DOI:** 10.3390/nu12010157

**Published:** 2020-01-06

**Authors:** Nguyen Hoang Anh, Sun Jo Kim, Nguyen Phuoc Long, Jung Eun Min, Young Cheol Yoon, Eun Goo Lee, Mina Kim, Tae Joon Kim, Yoon Young Yang, Eui Young Son, Sang Jun Yoon, Nguyen Co Diem, Hyung Min Kim, Sung Won Kwon

**Affiliations:** 1 College of Pharmacy, Seoul National University, Seoul 08826, Korea; 2018-23140@snu.ac.kr (N.H.A.); danielkim27@snu.ac.kr (S.J.K.); phuoclong@snu.ac.kr (N.P.L.); mje0107@snu.ac.kr (J.E.M.); yunyochl@snu.ac.kr (Y.C.Y.); piggypet@snu.ac.kr (E.G.L.); alsdk@snu.ac.kr (M.K.); joont@snu.ac.kr (T.J.K.); yyyang95@snu.ac.kr (Y.Y.Y.); dmldud3110@snu.ac.kr (E.Y.S.); supercanboy@snu.ac.kr (S.J.Y.); snuhmkim04@snu.ac.kr (H.M.K.); 2 School of Medicine, Vietnam National University, Ho Chi Minh City 70000, Vietnam; ncdiem.stu15@medvnu.edu.vn

**Keywords:** ginger, human health, randomized controlled trials, systematic review

## Abstract

Clinical applications of ginger with an expectation of clinical benefits are receiving significant attention. This systematic review aims to provide a comprehensive discussion in terms of the clinical effects of ginger in all reported areas. Following the preferred reporting items for systematic reviews and meta-analyses (PRISMA) guideline, randomized controlled trials on the effects of ginger were investigated. Accordingly, 109 eligible papers were fully extracted in terms of study design, population characteristics, evaluation systems, adverse effects, and main outcomes. The reporting quality of the included studies was assessed based on the Cochrane Collaboration’s tool for assessing the risk of bias in randomized trials and integrated together with studies that investigated the same subjects. The included studies that examined the improvement of nausea and vomiting in pregnancy, inflammation, metabolic syndromes, digestive function, and colorectal cancer’s markers were consistently supported, whereas other expected functions were relatively controversial. Nevertheless, only 43 clinical trials (39.4%) met the criterion of having a ‘high quality of evidence.’ In addition to the quality assessment result, small populations and unstandardized evaluation systems were the observed shortcomings in ginger clinical trials. Further studies with adequate designs are warranted to validate the reported clinical functions of ginger.

## 1. Introduction

Ginger (*Zingiber officinale* Roscoe), a well-known herbaceous plant, has been widely used as a flavoring agent and herbal medicine for centuries. Furthermore, the consumption of the ginger rhizome is a typical traditional remedy to relieve common health problems, including pain, nausea, and vomiting [1]. Notably, a prominent number of randomized clinical trials (RCTs) have been conducted to examine ginger’s antiemetic effect in various conditions such as motion sickness, pregnancy, and post-anesthesia [2,3,4]. More than approximately 100 compounds have reportedly been isolated from ginger [5]. Specifically, the major classes of ginger compounds are gingerol, shogaols, zingiberene, and zingerone, as well as other less common compounds, including terpenes, vitamins, and minerals [6]. Among them, gingerols are considered as the primary components, reported to possess several bioactivities [7]. As a result, many related biological activities have been explored such as those of antioxidant, antimicrobial, and anti-neuroinflammation, just to name a few [8]. Moreover, in recent years, the role of ginger has been extended to anticancer, chemotherapy-induced nausea and vomiting (CINV), and fatigue, as well as improvements in the quality of life in daily human work [9,10].

These potential pharmacological and physiological activities have led to a significant increase in the number of investigations on the health benefits of ginger. Regarding clinical aspects, there has been a trend of accumulative evidence in terms of ginger efficacy on human health. Indeed, a remarkable number of RCTs that have aimed to discover the benefits of ginger by reducing symptoms have been conducted. For example, multiple RCTs evaluated the effectiveness of ginger supplementation in reducing CINV in cancer patients, as well as in dysmenorrhea [11]. Moreover, several systematic reviews and meta-analysis (SR–MA), which aimed to assess the clinical ginger effectiveness, have been completed. In particular, Chen et al. conducted an SR–MA of oral ginger intake and found that ginger could effectively control menstrual pain in dysmenorrhea [11]. Another SR–MA study revealed that ginger improved lipid profiles and benefited the glucose control, insulin sensitivity, and glycosylated hemoglobin of type 2 diabetes mellitus [12]. In addition, ginger’s potency has been regularly proposed in arthritis, gastric dysfunction, and cancers [6,13,14].

Though several systematic reviews have been conducted, limitations regarding the reporting quality still persist. Important subjects that need further investigation include, but are not limited to, heterogeneous population, less stringent criteria, inadequate quality assessment, and inconsistent results. More importantly, there is still a lack of a comprehensive review in terms of critically assessing and comparing the quality of the evidence derived from RCTs in different domains of their efficacy. This study aimed to provide a systematic summarization of the effectiveness of oral ginger in human health and diseases in current RCTs. In addition, we evaluated ginger efficacy in every reported clinical-related aspect to provide future directions for the clinical research of ginger. Furthermore, we evaluated the ongoing development and achievement of ginger-related randomized clinical trials in specific representative topics. Finally, the shortcomings of available RCTs in terms of the ginger effect investigation were discussed.

## 2. Materials and Methods

### 2.1. Literature Search Strategy

The study follows the preferred reporting items for systematic reviews and meta-analyses (PRISMA) guidelines (Table S1) [15]. A systematic search was conducted on six libraries, including four English databases and two Korean databases. Firstly, for English literature, we searched Pubmed, EMBASE, Cochrane Central Register of Controlled Trials (CENTRAL), and Clinical Trials (ClinicalTrial.gov) with the query: “*Zingiber officinale*” OR “*Z. officinale*” OR “Ginger.” Next, two Korean databases, Korean studies Information Service System (KISS) and National Digital Science Library (NDSL), were searched using the query: “*Zingiber officinale*” OR “*Z. officinale*” OR “Ginger” and Korean language terms related to ginger. Data were collected up to July 2019 and regularly updated by manual search. There was no limitation in the search period.

### 2.2. Inclusion and Exclusion Criteria

We first imported the search results to Endnote X9 and performed the duplication removal step. The remaining papers were screened for title and abstract. This study focused on randomized clinical trials investigating the efficacy of ginger to improve human health as well as to support human disease. For that reason, any paper that reported the effectiveness of ginger in clinical aspects was included in this study. Inappropriate articles were excluded for the following reasons: (1) not clinical trials; (2) not related topic; (3) irrelevant data for analysis; (4) secondary analysis; (5) unavailable abstract or full-text; (6) duplication; and (7) case reports, letters, commentaries, meeting records, or review articles. At least two authors performed this step to evaluate the eligibility of each item. Next, the qualification of each paper was assessed by reading the full-text, and the qualified articles were collected for the next extraction data step. In addition, a manual search was also conducted by screening the reference lists of the selected articles. Finally, the qualified papers were included for the data extraction process.

### 2.3. Data Extraction

The details of demographic populations and study design information, including year, sample allocation, sample size, age, study design, symptom and disease, treatment and control group intervention information, duration of therapy, and blinding, were extracted. Importantly, we extracted the evaluation outcome system, key findings, and adverse effects of each study, as well as their found side effects, if any.

### 2.4. Quality Assessment

The Cochrane Collaboration’s tool was employed to evaluate the risk of bias in individual research for quality assessment [16]. The tool included seven items that aimed to evaluate the quality of study design (e.g., randomization), the result (e.g., outcome reporting), and other biases. All items were independently assessed and scored by at least two reviewers to avoid personal bias. Seven items were evaluated for all 109 studies, which were scored into three scales: H indicates a high risk of bias, U indicates an unclear risk of bias, and L indicates a low risk of bias. Additionally, each essential function was scored based on seven quality reporting items: 0 points for a high risk, 1 point for an unclear risk, and 2 points for a low risk of bias, and the quality assessment score (QA score) as the sum of each point. We set the criteria at the QA score of 10 or above as a ‘high quality of evidence.’

## 3. Results

### 3.1. Study Selection

**Figure 1:**
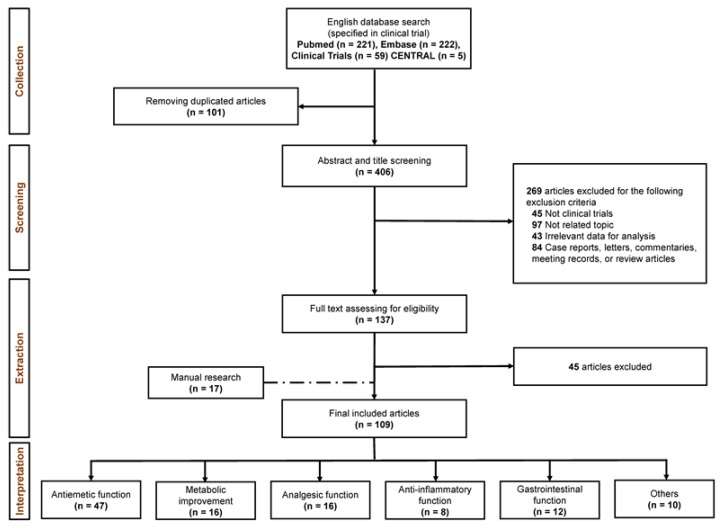
The workflow of systematical search on ginger randomized clinical trials (RCTs) with five categorized substantial functions.

After a systematical search that selectively focused on the study design of the clinical trials, we retrieved 221, 222, 59, and five papers from PubMed, Embase, Clinical Trials, and CENTRAL, respectively. Subsequently, 101 duplication records were eliminated. Next, the titles and abstracts of the remaining records were screened for eligibility for the extraction process. Finally, 137 records were included for the full-text monitoring, and 109 qualified articles, including manual search papers remained for the final data extraction step. Similarly, we extended our search method to two Korean literatures databases (KISS and NDSL) and retrieved a remarkable number of studies (n = 790 after duplication removal). Unfortunately, no Korean papers qualified after the standard flow of evaluation. The workflow of this study is shown in Figure 1.

### 3.2. Characteristics of Included Studies

**Figure 2:**
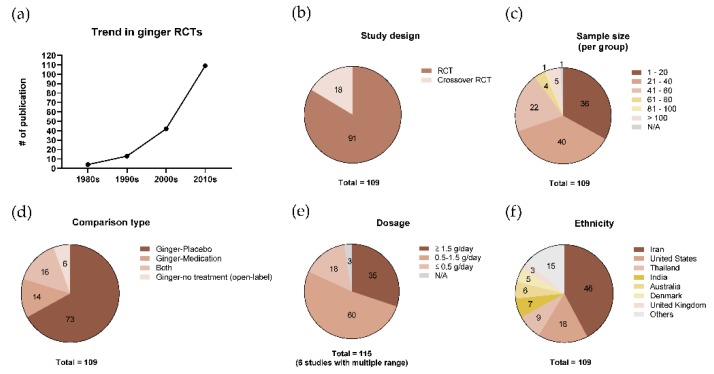
Features of ginger Randomized controlled trial (RCT) characteristics. (**a**) The trend in the publication of ginger RCTs over the decades, (**b**) the types of study design, (**c**) the ranges of pooled sample size per group, (**d**) the types of comparison in intervention, (**e**) the ranges of adopted dosage, and (**f**) the variety of ethnicity. N/A: Not available.

Demographics and RCT designs from the studies that were evaluated to have a ‘high quality of evidence’ are described in Table 1. The rest of the publications are described in Table S2 due to size of the sample. The trend in the publication of the included studies showed an apparent increase throughout the decades (Figure 2a). Eighteen studies were crossover trials that had a commonly known advantage in reducing the impact of confounding covariates (Figure 2b). Regarding sample size, a population of less than 60 participants per group was mostly conducted (Figure 2c). Seventy-three studies demonstrated the effect of ginger in comparison with placebo groups as a control, while 14 studies compared ginger with medication or other functional materials (Figure 2d). Sixteen studies were designed with both placebo and medication or other functional materials as the control groups (Figure 2d). A daily dosage of 0.5–1.5 g of ginger was frequently adopted, while six studies treated ginger with a multiple dosage range (Figure 2e). The ethnicity of participants in the included studies is summarized in Figure 2f, showing that more than half of the studies were conducted in Iran or the United States.

### 3.3. Clinical Effects of Ginger

The effects of ginger were reported in a variety of diseases and health conditions. In the following sections, we cover the five significant biological effects of ginger that were mainly examined in the included studies. In addition, other potential effects are also briefly summarized and discussed. Table 2 presents the key finding of each study introduced in Table 1. The key finding of other publications is provided in Table S3.

#### 3.3.1. Antiemetic Function

Major clinical trials with ginger were performed to evaluate its antiemetic activity (*n* = 47). Among these, CINV, a phenomenon induced by chemotherapeutic agents and which activates neurotransmitters as a side effect, was the most frequently investigated subject (*n* = 16). From 16 trials, eight demonstrated the positive effect of ginger treatment on the prevention and alleviation of CINV. Sanaati et al. reported that ginger significantly improved the quality of life in CINV group of patients who had received the first cycle of moderately to highly emetogenic chemotherapy compared to the placebo (median (interquartile range) = 124.5 (113.2, 126) vs. 111 (99, 126); *p* = 0.043) [18]. Furthermore, ginger effectively reduced acute and delayed CINV in both children and adults [56,57]. On the other hand, Thamlikitkul et al. and Li et al. concluded that ginger showed an insufficient effect on the prophylaxis of acute and delayed nausea and vomiting induced by an Adriamycin–cyclophosphamide regimen and a cisplatin regimen, respectively, which are highly emetogenic regimens [19,20].

Nausea and vomiting of pregnancy (NVP), also called hyperemesis gravidarum in severe cases, is a symptom that commonly occurs in pregnant women and has the potential to cause nutritional deficiency. There were 14 clinical trials regarding the alleviative effect of ginger on NVP. Eight studies investigated the antiemetic effect of ginger by comparing it to a placebo group, revealing significant effects in the ginger treatment group. Simultaneously, ginger showed a similar effect compared to other medication groups, such as vitamin B6 (pyridoxine), antihistamine, or metoclopramide. However, a study by Ensiyeh et al. concluded that ginger is more effective in relieving the severity of nausea compared to vitamin B6 (*p* = 0.024) [3].

Postoperative nausea and vomiting (PONV) is an emetic event that is induced in the patients after surgical procedures, and it is mainly caused by the anesthetic. Ginger treatment was used as an intervention in eleven RCTs. However, five RCTs concluded that there were no significant results with ginger.

Still, ginger may have a beneficial effect in gynecological patients, according to the results from Apariman et al., Chaiyakunapruk et al., Phillips et al., and Bone et al. [58,59,60,61]. Finally, in the study by Dabaghzadeh et al., the researchers primally examined the effect of ginger and demonstrated its benefit on the prevention of nausea and vomiting induced by an antiretroviral regimen (*p* = 0.001) [62]. Efficacy on motion sickness and vertigo was also examined in several studies, with results indicating different aspects between the studies.

#### 3.3.2. Gastrointestinal Function

As an extension of the antiemetic property, ginger has been studied for its protective effect on the gastrointestinal system. Seven RCTs examined ginger’s effect on gastric function, mostly regarding gastric emptying and dysrhythmia. All studies that observed gastric emptying rate reported ginger as a digestive enhancer, except the study from Phillips et al., where they denied the facilitation of gastric function as demonstrated by the paracetamol absorption rate [50]. Lien et al. reported that ginger treatment significantly reduced tachygastric activity induced by circular vection, a phenomenon of feeling a rotating sensation without actual movement, in a rotating drum (*p* < 0.05). Gonlachanvit et al. investigated the beneficial effect of the ginger root in the prevention of slow-wave dysrhythmias induced by acute hyperglycemic events (*p* < 0.05) [63,64].

Four RCTs examined the anticancer effect of ginger, all of which evaluated the risk of colorectal cancer according to the ginger treatment. Collectively, ginger has a beneficial effect on colorectal cancer by reducing tumorigenic risk factors. However, Jiang et al. reported that participants with an average risk of colorectal cancer showed no significant aspect between the ginger and placebo groups [14]. Citronberg et al. investigated cell cycle markers with biopsies from the patients with increased risk of colorectal cancer and demonstrated the regulation of apoptotic and differentiation markers by ginger supplementation [51]. Lastly, one study from Miranda et al. examined the symptomatic relief on irritable bowel syndrome patients upon ginger application and found no evidence in the reduction of symptoms (*p* > 0.05) [54].

#### 3.3.3. Analgesic Function

Seven RCTs examined the effect of ginger on primary dysmenorrhea. Four trials compared the analgesic effect with other medications such as mefenamic acid, ibuprofen, and zinc sulfate, which displayed similar efficiencies to ginger. Three trials adopted a placebo as a control group, reporting the reduction of pain by the level of visual analog scale. For example, Rahnama et al. reported that ginger significantly improved primary dysmenorrhea in ginger-treated patients for five days, beginning two days before the onset of menstruation [45]. Only one study concluded that ginger was an insufficient pain reliever upon comparison with stretching and exercising for alleviation [65]. 

Four RCTs administered ginger to a group of participants with muscular pain, with varied result observed. Two studies reported a lack of evidence regarding the effect of ginger, and the other two reported that ginger partially attenuated muscular pain compared to the placebo group. Migraines and headaches were examined to assess the pain-relieving attributes of ginger in three RCTs. Maghbooli et al. and Martins et al. compared the effects of sumatriptan and placebo, respectively, both showing there was a significant difference in symptom attenuation (*p* < 0.05) [23,66]. Patients with low back pain and chest pain caused by percutaneous transluminal coronary angioplasty were also selected to assess the analgesic effects of ginger, and both studies concluded that ginger was a useful option for pain relief.

#### 3.3.4. Inflammatory Effect

Overall, eight RCTs reported the anti-inflammatory effect of ginger supplementation. Among them, arthritis-related diseases were the most conducted studies, particularly osteoarthritis (OA). Regarding OA, six studies investigated the efficiency of the constituents of ginger that serve as anti-inflammatory agents. All studies reported improvement following ginger intake compared to the control group. For instance, Mozaffari-Khosravi et al. proposed that benefits of ginger were observed due to a reduction in the level of the proinflammatory cytokines after three months of consuming 500 mg of ginger powder [13]. Other studies showed a promising benefit of ginger in relieving pain in OA patients. Furthermore, no significant adverse effects were observed during the trials. An additional study that assessed ginger’s effects on rheumatoid arthritis demonstrated improvement by reducing symptoms via inducing *FOXP3* gene expression. Finally, Kulkarni et al. reported that ginger supplementation alone and combined with antitubercular treatment significantly helped to decrease tumor necrosis factor (TNF) alpha, ferritin, and malondialdehyde (MDA) levels compared to the control group [67].

#### 3.3.5. Metabolic Improvement

Studies evaluating the efficiency of ginger in metabolic syndromes have also been widely conducted. Most of the included studies assessed the association of type 2 diabetes mellitus (T2DM) and obesity with ginger supplementation. In detail, five studies explored the effect of ginger on the diabetes-related indices such as glycemic markers, lipid level, and blood pressure, while four studies focused on various conditions related to obesity such as cardiovascular disease, serum adipocytokines, and breast cancer. For example, three studies evaluated the influence of ginger on biochemical parameters related to T2DM and demonstrated the significant lowering of fasting blood sugar, hemoglobin A1c (HbA1c), insulin sensitivity, and insulin resistance. Furthermore, lipid profile, inflammatory markers, and antioxidants were also affected by ginger intake, which was demonstrated by the reduction of the C-reactive protein, triglycerides (TG), low-density lipoprotein cholesterol (LDL-C), and malondialdehyde. Another study assessed the relationship between ginger and blood pressure in T2DM but showed no significant differences compared to the control group. Regarding obesity, the included study targeted the effect of ginger on obesity. In obese women, Attari et al. reported that ginger supplements had a minor benefit on weight loss, the reduction of insulin and homeostasis model assessment of insulin resistance (HOMA-IR), and the increasing of quantitative insulin sensitivity check index (QUICKI) [25]. In terms of obesity-related cardiovascular risk factors, ginger was reported to be beneficial in lowering the risk factors, such as body fat mass, body fat percentage, total cholesterol, waist circumference, waist-to-hip ratio, and insulin resistance. In addition, ginger was suggested to have antioxidant and anti-dysmetabolic effects in obese women with breast cancer. Finally, other studies aimed to explore the effect of ginger on lipid metabolism including fat utilization and triglyceride-lowering efficacy. In general, ginger was believed to provide potential benefits by reducing the risk factors of metabolic syndromes. Moreover, no serious adverse effects were observed in all included studies.

#### 3.3.6. Other Clinical Functions

Besides the effects introduced above, several different functions, such as thermoregulatory, thrombotic, and respiratory function, were evaluated at the clinical level. The thermogenetic function of ginger was examined by three randomized crossover trials, and only one study observed the expected outcome. In terms of respiratory function, acute respiratory distress syndrome (ARDS) and asthma were examined to evaluate the improvement of symptoms. Ginger effectively reduced the duration of mechanical ventilation and the length of stay in the intensive care unit in ARDS patients; it also improved asthmatic symptoms. There were three studies regarding thrombotic function, and two studies reported that ginger had little effect on the thrombotic reaction. However, Bordia et al. reported that a single dose of 10 g of powdered ginger significantly reduced Adenosine diphosphate-induced and epinephrine-induced platelet aggregation in patients who were recovering from myocardial infarction [68]. Kashefi et al. administered ginger to 15–18 years old patients with heavy menstrual bleeding, and the ginger treatment group demonstrated a significant reduction in menstrual blood loss (*p* < 0.001) [28]. Paritakul et al. examined the effect of ginger on breast milk production in a group of women after delivery and concluded that ginger treatment significantly increased milk volume on the third day postpartum compared to the placebo (*p* < 0.01) [29].

### 3.4. Adverse Effects

Seventeen studies provided information about adverse effects in their research papers, most of which were not considered to be severely harmful to the participants. Among the adverse effects, gastrointestinal-related symptoms were mostly reported to reverse the gastrointestinal protective effect of ginger in other aspects. Heartburn, a general symptom of gastroesophageal reflux disease, was reported in sixteen studies. Five studies reported nausea as a side effect of ginger treatment, which was the primary topic evaluated to observe the clinical effect of ginger. Diarrhea was reported in two studies in groups of patients with heavy menstrual bleeding and after elective cesarean section. Other GI symptoms included abdominal pain, bloating, gas, and epigastric distress. Furthermore, cardiovascular symptoms and respiratory symptoms were observed in a ginger-treated patient group who underwent laparoscopic surgery. The types of adverse effects, incidence rate, and dosage are described in Table S4.

### 3.5. Quality Assessment

**Figure 3:**
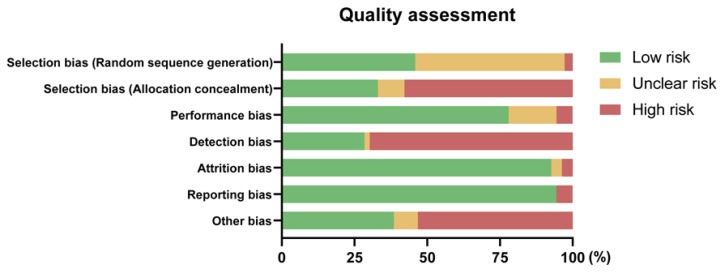
Methodological quality graph: The risk of bias for each item is expressed by percentage.

Firstly, regarding selection bias, three studies were judged as having a high risk of bias in random sequence generation, and 50 studies that were assigned to low risk described the methodological procedure of randomization. Thirty-six studies described the allocation concealment method with detailed explanations, and ten studies that provided ambiguous information about allocation were assigned to having an unclear risk. Sixty-three were judged as having a high risk of bias in allocation concealment. For example, in the study of Shirvani et al., participants were divided into a ginger treatment group and an exercising group, meaning that the allocation was easily predictable [65]. Second, in an aspect of performance bias, blinding was a common practice in more than 75% of the studies (*n* = 85). Eighteen studies were judged as showing an unclear risk, as they did not mention blinding or similarity of the administration appearance. The majority of studies did not report about the blinding of outcome assessment and were assigned to have a high risk in detection bias (*n* = 75, 69.7%). Thirty-one papers with low risk on this item described the blinding of the data analyzer or stated that their study design was triple-blinded. Attrition bias and reporting bias were the two least potential biases that could interrupt the results of the included trials, where 101 and 103 studies were assigned to low risks of attrition and reporting bias, respectively. Finally, 42 studies described the limitations of their studies and implied the possibility of any other biases, whereas 58 studies did not mention limitations and were assigned as a high risk. For better visualization, a methodological quality graph is introduced in Figure 3.

**Figure 4:**
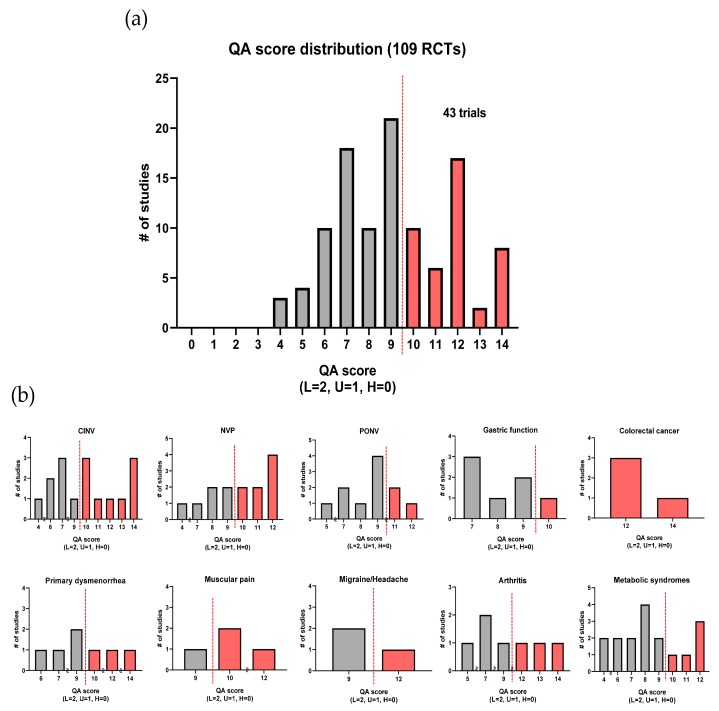
Distribution of ginger randomized controlled trials (RCTs) based on quality assessment (QA) score: a bright color indicates a ‘high quality of evidence,’ whereas a dark color indicates the opposite. (**a**) In total, 43 RCTs were addressed to have a high quality of evidence, and (**b**) each important function was evaluated (CINV: chemotherapy-induced nausea and vomiting; NVP: nausea and vomiting of pregnancy; PONV: postoperative nausea and vomiting).

In addition to investigating each quality assessment item for overall studies, we also compared the quality of each important function based on our quality assessment scoring system, as described in Figure 4. The quality assessment (QA) outcomes and the QA score for each study are provided in Table S5.

## 4. Discussion

The clinical applications of ginger as a medicinal or adjuvant therapy have been receiving significant attention due to its several expected functions, general use globally, and empirically guaranteed safety [69,70]. However, an overall comparison of the studies dealing with different functional effects and studies examining minor functions has yet to be adequately performed. As demonstrated by our assessment on reporting quality, attrition bias, and reporting bias were observed to be minor in a majority of studies, and the blinding of participants was also relatively well managed. However, several studies did not describe the method for random sequence generation, method of concealment in allocating procedure, and the blinding of assessors in detail. As a result, several ginger clinical studies had a substantial likelihood of selection and detection bias. For example, in a study by Vladimir et al., the ginger-treated group and the diclofenac-treated group received different formulations of drugs, which might have provided information about allocation with high probability [71]. Finally, only eight studies were judged as having low risks for every type of bias based on the Cochrane Collaboration’s quality assessment tool [14,18,20,28,29,31,45,55]. Regarding the reporting quality of essential functions, 43 RCTs out of 109 (39.4%) reported a high quality of evidence. Collectively, no major function satisfied the criteria for every study except colorectal cancer. Major functions that indicated a high quality of evidence in more than half of their studies were CINV, NVP, colorectal cancer, and muscular pain. Less than one-third of PONV, gastric function and metabolic syndrome investigations were appraised to have a high quality of evidence.

Only 17 the included RCTs (15.6%) presented adverse response information. There were no life-threatening or severe cases reported. Heartburn was the only symptom consistently reported in 16 studies where the participants received between 500 and 2000 mg of ginger per day. This result was supported by the biological property of ginger’s constituents inhibiting cyclooxygenase, which has a role in gastric mucosal defense [9,72]. Other reported symptoms could not be generalized as side effects of ginger because the number of reporting studies and the number of participants in each study were both small. For example, a study by Kashefi et al. reported diarrhea as an adverse event in the ginger treatment group. However, only one out of 46 patients (2.17%) reported the symptom, indicating no statistically significant correlation to ginger [28]. Moreover, the ratio of reported unexpected cases per group varied considerably between studies. A methodological description regarding the evaluation system used for adverse effects should be provided in future studies for accurate data integration.

Ginger had been investigated as an additional or alternative treatment to standard regimens in fifteen clinical trials of CINV, but the results between studies were controversial, with only about half of them demonstrating significant effects corresponding to the results of previous systematic reviews [73,74]. Moreover, studies from Yunes et al. and Müzeyyen et al., where they concluded the antiemetic effect of ginger, were assessed to have a high risk of bias in the blinding of participants [75,76]. The dosage of ginger varied among the studies without any correlation with outcomes. Hence, appropriately designed ginger clinical studies on CINV need to be performed for apparent demonstration. On the contrary, ginger showed a promising effect on NVP. All 10 RCTs on NVP concluded that ginger is as effective as other antiemetic medications such as pyridoxine, metoclopramide or dimenhydrinate or more effective than the placebo at least in some aspects with a dosage less than 1.5 g. An observation study from Portnoi et al. reported that a group of pregnant women showed no statistical differences in teratogenicity when compared to a group receiving nonteratogenic drugs [77]. A large population-based cohort from the Norwegian Mother and Child Cohort study correspondingly reported that ginger did not affect the risk of teratogenicity and abnormalities in birth [78]. Moreover, a review from Stanisiere et al. found that there were no severe side effects with ginger consumption in controlled, uncontrolled, and pre-clinical studies, with a significant reduction in nausea and vomiting [79]. These results support the idea that ginger may be an alternative approach for antiemetic therapy in women during the gestation period of pregnancy. The effectiveness of ginger on PONV, similar to the results from CINV, was inconsistent between studies even with a comparatively high dose (2 g), indicating that further investigations are imperative [60,80,81].

Studies on digestive function mostly reported a positive effect of ginger in reducing gastric emptying time and dysrhythmia. However, symptoms and evaluation methods varied among studies, with substandard general qualities, making it challenging to integrate outcomes. Investigations on the anticancer activity of ginger or its active constituents, especially shogaols and gingerol species, have been conducted in various types of cancer with different models [6,82]. However, there was no direct evaluation of the incidence or survival of patients in the four trials that evaluated ginger’s effect on colorectal cancer. Instead, factors of inflammation, proliferation, differentiation, and apoptosis were measured upon treatment with 1–2 g of ginger to evaluate the improvement of risk. All four studies reported that colorectal cancer-related risk factors were decreased in the ginger treatment group. Even though only four studies were assessed for this review in regard to colorectal cancer, the results were promising, and the quality of the studies was high, with a low possibility of any bias. Accordingly, further clinical trials are imperative to reinforce the colorectal cancer-preventive effect of ginger.

The analgesic effect of ginger was primarily examined on the alleviation of primary dysmenorrhea. Six studies reported that ginger improved pain relief and had similar effectiveness with medications such as mefenamic acid and ibuprofen compared to the placebo group, except for a study from Marjan et al., where ginger was reported to have no benefit [65]. However, the comparison group in this study managed dysmenorrhea with exercise instead of a placebo or other medication, thus giving a high risk of confounding factors. Furthermore, the dosage was 250 mg a day, whereas the treatment dose ranged between 750 and 1500 mg a day in the other six studies. Altogether, the analgesic effect of ginger on primary dysmenorrhea is worthy of consideration, but more appropriately designed studies should be conducted because the overall quality of the studies was not high. Other types of pain were also improved with ginger treatment in most of the studies. This may be an indicative result from the previously known mechanisms of ginger’s active compounds to suppress cyclooxygenase and lipoxygenase [9,72].

The effectiveness of ginger in anti-inflammation and metabolic syndromes has been well studied. For instance, it has been found to significantly reduce symptoms in patients with arthritis-related diseases. Nevertheless, the benefit of ginger among the studies was inconsistent in terms of effectiveness. Noticeably, several studies compared the effectiveness of ginger with other anti-inflammatory drugs. One study concluded that ginger is as effective as ibuprofen in reducing the symptoms of OA, while another study reported the opposite result [83,84]. However, all the included studies were conducted with a sample size of less than 100 participants in each group. In addition, the dosages among the studies varied from 15 to 750 mg, and the treatment duration ranged from three weeks to 12 weeks. Therefore, further studies with larger sample sizes and standardized study designs should be conducted to confirm the effect of ginger on the symptoms of OA. Regarding metabolic diseases, many studies have demonstrated that ginger can improve blood biochemical parameters and lipid profiles, which can additionally help in reducing the risk of cardiovascular diseases. For instance, ginger supplementation has been found to notably reduce fasting blood sugar, HbA1c, and insulin resistance [85,86,87]. Additionally, lipid profiles (e.g., total cholesterol, and LDL-C), C-reactive protein, obesity-related cardiovascular risk factors have been found to reduce with ginger intake [24,88,89,90]. In general, ginger has been found to indicate a beneficial effect on high dosage and long-term treatment in metabolic diseases. However, an obvious limitation is that all studies were performed in a sample size of fewer than 50 participants. Further investigations should be conducted to validate the effect of ginger on metabolic syndrome.

Additionally, the shortcomings in the current ginger clinical trials across domains need to be specified. First, the group size was generally small and rarely exceeded 100 participants per group (only five studies) [30,37,80,91,92]. Thirty-six studies conducted clinical trials group sizes of less than 20 patients. Therefore, the drawn conclusions from these studies possess the risk of being underpowered. Secondly, the evaluation systems of some symptoms varied between studies that focused on a similar subject. For example, the beneficial effect on digestive and colorectal cancer-preventive function was consistently reported in related studies. However, the different evaluation parameters and markers made it challenging to integrate the outcomes. Finally, we cannot rule out the possibility of low external validity due to the diversity in ethnicity: There were 46 trials from Iran and 18 trials from the United States.

## 5. Conclusions

Ginger is a natural spice that is used in diverse regions to add a pungent flavor to food. Furthermore, ginger has been used as an herbal medicine for common health problems. This systematic review is the first study that has exclusively collected RCTs regarding the efficiency of ginger in several human health conditions. The clinical effects of ginger have been introduced as six subsections: nausea and vomiting, gastrointestinal function, pain, inflammation, metabolic syndromes, and other symptoms. Reportedly, ginger has been effective in a majority of studies, including those that examined the alleviation of NVP, digestive function, improvement in the expression level of markers for colorectal cancer risk, and anti-inflammatory functions. Several other functions have also been regarded as beneficial in trials, with some confronting results. However, a few drawbacks regarding the quality of the trials, inconsistent evaluation systems or parameters, and the generally small size of the studies need to be noted. Therefore, systematically designed research with detailed descriptions of methodology and a sufficient pool of participants is necessary for future clinical trials to address the functional characteristics of ginger.

## Figures and Tables

**Table 1:** Study design and demographic description of the included studies with a “high quality of evidence” (quality assessment score is of at least 10).

Author (Year)	Cohort Allocation	Study Design	Type of Disease/Symptom	Intervention			Comparator			Duration	Blind
				Number	M/F	Dosage	Number	M/F	Dosage		
Marx et al. (2017) [17]	Australia	Randomized controlled trial	Chemotherapy-induced nausea and vomiting	24	8/16	300 mg of ginger extract (5% gingerol)/cap, 4 capsules/day	27	11/16	300 mg of placebo/cap, 4 capsules/day	5 days	Double-blind
Sanaati et al. (2016) [18]	Iran	Randomized controlled trial	Chemotherapy-induced nausea and vomiting	15	0/15	500 mg of powdered ginger/cap, 2 capsules/day and DMA regimen *	15	0/15	DMA regimen *	5 days before and 5 days after chemotherapy	Double-blind
	Iran	Randomized controlled trial	Chemotherapy-induced nausea and vomiting	15	0/15	500 mg of powdered ginger/cap, 2 capsules/day and DMA regimen *	15	0/15	500 mg of powdered chamomile/cap, 2 capsules/day and DMA regimen *	5 days before and 5 days after chemotherapy	Double-blind
Thamlikitkul et al. (2016) [19]	Thailand	Crossover randomized controlled trial	Chemotherapy-induced nausea and vomiting	Second cycle: 19; Third cycle: 15	Second cycle: 0/19 Third cycle: 0/15	500 mg of powdered ginger/cap, 2 capsules/day	Second cycle: 15; Third cycle: 19	Second cycle: 0/15; Third cycle: 0/19	500 mg of placebo/cap, 2 capsules/day	5 days at each second and third cycle of chemotherapy	Double-blind
Li et al. (2017) [20]	China	Randomized controlled trial	Chemotherapy-induced nausea and vomiting	71	53/18	250 mg of powdered ginger (5% gingerols)/cap, 2 capsules/day, bid	69	47/22	250 mg of corn starch/cap, 2 capsules/day, bid	5 days	Double-blind
Ansari et al. (2016) [21]	Iran	Randomized controlled trial	Chemotherapy-induced nausea and vomiting	57	0/57	250 mg of powdered ginger/cap, 4 capsules/day, bid	62	0/62	250 mg of starch/cap, 4 capsules/day, bid	3 days for each 3 cycles	Single-blind
Sharifzadeh et al. (2018) [4]	Iran	Randomized controlled trial	Nausea and vomiting because of pregnancy	28	0/28	500 mg of ginger/cap, 2 capsules/day	26	0/26	40 mg of vitamin B6/cap, 2 capsules/day	4 days	Triple- blind
	Iran	Randomized controlled trial	Nausea and vomiting because of pregnancy	28	0/28	500 mg of ginger/cap, 2 capsules/day	23	0/23	2 placebo capsules/day	4 days	Triple-blind
Matsumura et al. (2015) [22]	United States	Randomized controlled trial	Muscle damage and delayed onset muscle soreness	10	5/5	4 g of powdered ginger in capsule(s)/day	10	5/5	4 g of dextrose in capsule(s)/day	5 days	Double-blind
Martins et al. (2018) [23]	Brazil	Randomized controlled trial	Migraine	30	4/26	200 mg of ginger extract/cap, 2 capsules/dose + 100 mg of ketoprofen (i.v.)	30	4/26	200 mg of cellulose/cap, 2 capsules/dose + 100 mg of ketoprofen (i.v.)	Single dose	Double-blind
Arzati et al. (2017) [24]	Iran	Randomized controlled trial	Type 2 diabetes mellitus	25	9/16	500 mg of ginger/cap, 4 capsules/day	25	7/18	500 mg of wheat flour/cap, 4 capsules/day	10 weeks	Double-blind
Attari et al. (2016) [25]	Iran	Randomized controlled trial	Obesity	39	0/39	1 g of powdered ginger/tab, 2 tablets/day	31	0/31	1 g of corn starch and other excipients/tab, 2tablets/day	12 weeks	Double-blind
Attari et al. (2015) [26]	Iran	Randomized controlled trial	Obesity management	39	0/39	1 g of powdered ginger/tab, 2 tablets/day	31	0/31	1 g of corn starch/tab, 2 tablets/day	12 weeks	Double-blind
Mozaffari-Khosravi et al. (2016) [13]	Iran	Randomized controlled trial	Knee osteoarthritis	50	3/47	500 mg of powdered ginger/cap, 2 capsules/day	50	7/43	500 mg of starch/cap, 2 capsules/day	3 months	Double-blind
Aryaeian et al. (2019) [27]	Iran	Randomized controlled trial	Active rheumatoid arthritis	33	4/29	750 mg of powdered ginger/cap, 2 capsules/day	30	3/27	750 mg of wheat flour/cap, 2 capsules/day	12 weeks	Double-blind
Kashefi et al. (2015) [28]	Iran	Randomized controlled trial	Heavy menstrual bleeding	43 (1st month); 41 (2nd month); 38 (3rd month)	0/43 (1st month) 0/41 (2nd month) 0/38 (3rd month)	250 mg of powdered ginger/cap, 3 capsules/day	43 (1st month); 38 (2nd month); 33 (3rd month)	0/43 (1st month) 0/38 (2nd month) 0/33 (3rd month)	250 mg of lactose/cap, 3 capsules/day	From the day before menstrual bleeding to the 3rd day of the menstrual period	Double-blind
Paritakul et al. (2016) [29]	Thailand	Randomized controlled trial	Breast milk volume of postpartum women who delivered a term baby (≥37 weeks of gestation)	15	0/15	500 mg of powdered ginger/cap, 2 capsules/day	21	0/21	500 mg of corn starch/cap, 2 capsules/day	7 days	Double-blind
Ryan et al. (2012) [30]	United States	Randomized controlled trial	Chemotherapy-induced nausea and vomiting	134	12/122	One ginger capsule (250 mg of ginger extract) + 2 placebo capsules, twice/day	149	14/135	Three placebo capsules, twice/day	6 days	Double-blind
	United States	Randomized controlled trial	Chemotherapy-induced nausea and vomiting	141	19/122	Two ginger capsules (250 mg of ginger extract) + 1 placebo capsule, twice/day					
	United States	Randomized controlled trial	Chemotherapy-induced nausea and vomiting	152	10/142	Three ginger capsules (250 mg of ginger extract), twice/day					
Zick et al. (2008) [31]	United States	Randomized controlled trial	Chemotherapy-induced nausea and vomiting	53	14/39	250 mg of dry ginger root extract/cap, 4 ginger capsules and 4 lactose capsules/day	57	14/43	250 mg of lactose/cap, 8 capsules/day	28 days	Double-blind
	United States	Randomized controlled trial	Chemotherapy-induced nausea and vomiting	52	12/40	250 mg of dry ginger root extract/cap, 8 capsules/day					
Fahimi et al. (2010) [32]	Iran	Crossover randomized controlled trial	Chemotherapy-induced nausea and vomiting	36	26/10	250 mg of powdered ginger/cap, 2 capsules/day	36	26/10	250 mg of lactose/cap, 2 capsules/day	3 days for each period	Double-blind
Yekta et al. (2012) [33]	Iran	Randomized controlled trial	Chemotherapy-induced nausea and vomiting	40	0/40	250 mg of powdered ginger/cap, 4 capsules/day	40	0/40	250 mg of starch/cap, 4 capsules/day	6 days from three days before a chemotherapy session	Double-blind
Ensiyeh et al. (2009) [3]	Iran	Randomized controlled trial	Nausea and vomiting because of pregnancy	35	0/35	500 mg of powdered ginger/cap, 2 capsules/day	34	0/34	20 mg of vitamin B6/cap, 2 capsules/day	4 days	Double-blind
Willetts et al. (2003) [34]	Australia	Randomized controlled trial	Nausea and vomiting because of pregnancy	48	0/48	125 mg of ginger extract/cap, 4 capsules/day	51	0/51	4 capsules/day, each capsule containing soya bean oil	4 days	Double-blind
Vutyavanich et al. (2001) [35]	Thailand	Randomized controlled trial	Nausea and vomiting because of pregnancy	32	0/32	250 mg of powdered ginger/cap, 4 capsules/day	38	0/38	4 placebo capsules/day	4 days	Double-blind
Fischer-Rasmussen et al. (1990) [36]	Denmark	Crossover randomized controlled trial	Nausea and vomiting because of pregnancy	27	0/27	250 mg of powdered ginger root/cap, 4 capsules/day	27	0/27	250 mg of lactose/cap, 4 capsules/day	Two periods of 4 days	Double-blind
Smith et al. (2004) [37]	Australia	Randomized controlled trial	Nausea and vomiting because of pregnancy	145	0/145	350 mg of ginger/cap, 3 capsules/day, tid	146	0/146	25 mg of vitamin B6/cap, 3 capsules/day, tid	3 weeks	Double-blind
Biswas et al. (2011) [38]	India	Randomized controlled trial	Nausea and vomiting because of pregnancy	34	0/34	150 mg of dried ginger extract/tab, 3 tablets/day	29	0/29	10 mg of doxylamine + 10 mg of pyridoxine/tab, 2 or 3 tablets/day	3 weeks	Single-blind
Firouzbakht et al. (2014) [39]	Iran	Randomized controlled trial	Nausea and vomiting because of pregnancy	24	0/24	250 mg of powdered ginger/cap, 4 capsules/day	35	0/35	40 mg of vitamin B6/cap, 4 capsules/day	4 days	Double-blind
	Iran	Randomized controlled trial	Nausea and vomiting because of pregnancy	24	0/24	250 mg of powdered ginger/cap, 4 capsules/day	28	0/28	40 mg of sugar/cap, 4 capsules/day	4 days	Double-blind
Arfeen et al. (1995) [40]	Australia	Randomized controlled trial	Postoperative nausea and vomiting	36	N/A	One capsule containing 500 mg of powdered ginger and one placebo capsule	36	N/A	Two placebo capsules	Single-dose	Double-blind
	Australia	Randomized controlled trial	Postoperative nausea and vomiting	36	N/A	Two capsules containing 500 mg of powdered ginger	36	N/A	Two placebo capsules	Single-dose	Double-blind
Eberhart et al. (2003) [41]	Germany	Randomized controlled trial	Postoperative nausea and vomiting	59	0/59	100 mg of ginger extract/cap, 1 ginger capsule + 1 placebo capsule/dose	59	0/59	2 placebo capsules/dose	Triple dose: before operation and 3 h and 6 h post operation	Double-blind
	Germany	Randomized controlled trial	Postoperative nausea and vomiting	57	0/57	100 mg of ginger extract/cap, 2 ginger capsules/dose	59	0/59	2 placebo capsules/dose	Triple dose: before operation and 3 h and 6 h post operation	Double-blind
Mandal et al. (2014) [42]	India	Randomized controlled trial	Postoperative nausea and vomiting	50	42/8	0.5 g of powdered ginger/cap, 2 capsules/dose + 4 mg of ondansetron (i.v.)	50	38/12	2 capsules of placebo/dose + 4 mg of ondansetron (i.v.)	1 h before induction of general anesthesia	Double-blind
Ozgoli et al. (2009) [43]	Iran	Randomized controlled trial	Primary dysmenorrhea	50	0/50	250 mg of powdered ginger/cap, 4 capsules/day	50	0/50	250 mg of mefenamic acid/cap, 4 capsules/day	3 days	Double-blind
	Iran	Randomized controlled trial	Primary dysmenorrhea	50	0/50	250 mg of powdered ginger/cap, 4 capsules/day	50	0/50	400 mg of ibuprofen/cap, 4 capsules/day	3 days	Double-blind
Kashefi et al. (2013) [44]	Iran	Randomized controlled trial	Primary dysmenorrhea	47 (1st month); 45 (2nd month)	0/47 (1st month) 0/45 (2nd month)	250 mg of powdered ginger/cap, 3 capsules/day	54 (1st month); 53 (2nd month)	0/54 (1st month); 0/53 (2nd month)	220 mg of zinc sulfate/cap, 3 capsules/day	4 days	N/A
	Iran	Randomized controlled trial	Primary dysmenorrhea	47 (1st month); 45 (2nd month)	0/47 (1st month) 0/45 (2nd month)	250 mg of powdered ginger/cap, 3 capsules/day	45 (1st month); 42 (2nd month)	0/45 (1st month); 0/42 (2nd month)	220 mg of lactose/cap, 3 capsules/day	4 days	N/A
Rahnama et al. (2012) [45]	Iran	Randomized controlled trial	Primary dysmenorrhea	59	0/59	500 mg of powdered ginger root/cap, 3 capsules/day	59	0/59	500 mg of toast powder (placebo)/cap, 3 capsules/day	From two days before the onset of menstrual period to first three days of menstrual period	Double-blind
	Iran	Randomized controlled trial	Primary dysmenorrhea	59	0/59	500 mg of powdered ginger root/cap, 3 capsules/day	46	0/46	500 mg of toast powder (placebo)/cap, 3 capsules/day	First three days of the menstrual period	Double-blind
Black et al. (2010) [46]	United States	Crossover randomized controlled trial	Muscle pain, inflammation, and dysfunction induced by eccentric exercise	27	12/15	Six capsules containing 2 g of dried ginger extract with 250 mL of water and one tablespoon of olive oil.	27	12/15	Six capsules containing 2 g of flour with 250 mL of water and one tablespoon of olive oil.	Single-dose	Double-blind
Black et al. (2009) [47]	United States	Randomized controlled trial	Muscle pain caused by eccentric exercise	Raw ginger study: 17	Raw ginger study: 3/14	Raw ginger study: 2 g of raw ginger /day	Raw ginger study: 17	Raw ginger study: 3/14	Raw ginger study: 2 g of yellow cornflower/day	11 days	Double-blind
	United States	Randomized controlled trial	Muscle pain caused by eccentric exercise	Heat-treated ginger study: 20	Heat-treated ginger study: 7/13	Heat-treated ginger study: 2 g of heat-treated ginger/day	Heat-treated ginger study: 20	Heat-treated ginger study: 7/13	Heat-treated ginger study: 2 g of powdered brown sugar/day	11 days	Double-blind
Mahluji et al. (2013) [48]	Iran	Randomized controlled trial	Type 2 diabetes mellitus	28	N/A	1 g of powdered ginger/tab, 2 tablets/day	30	N/A	1 g of corn starch/tab, 2 tablets/day	8 weeks	Double-blind
Khandouzi et al. (2013) [49]	Iran	Randomized controlled trial	Type 2 diabetes mellitus	22	5/17	1 g of powdered ginger/cap, 2 capsules/day	19	9/10	Lactose (placebo)	12 weeks	Double-blind
Phillips et al. (1992) [50]	United Kingdom	Crossover randomized controlled trial	Gastric emptying	16	N/A	500 mg of powdered ginger/cap, 2 capsules/dose	16	N/A	500 mg of lactose/cap, 2 capsules/dose	Single-dose	Double-blind
Jiang et al. (2013) [14]	United States	Randomized controlled trial	Normal risk for colorectal cancer	14	N/A	250 mg of ginger extract/cap, 8 capsules/day	16	N/A	Placebo (lactose)	28 days	Double-blind
	United States	Randomized controlled trial	High risk for colorectal cancer	10	4/6	250 mg of ginger extract/cap, 8 capsules/day	10	3/7	Placebo (lactose)	28 days	Double-blind
Citronberg et al. (2013) [51]	United States	Randomized controlled trial	Cell cycle biomarkers in the normal-appearing colonic mucosa of patients at increased risk for colorectal cancer	10	4/6	250 mg of ginger extract powder (5% gingerols)/cap, 8 capsules/day, bid	10	3/7	250 mg of lactose powder /cap, 8 capsules/day, bid	28 days	Double-blind
Zick et al. (2011) [52]	United States	Randomized controlled trial	Eicosanoids level of patients with normal risk for colorectal cancer	16	N/A	250 mg of dry ginger root extract/cap, 8 capsules/day	17	N/A	250 mg of lactose/cap, 8 capsules/day	28 days	Triple-blind
Zick et al. (2014) [53]	United States	Randomized controlled trial	Eicosanoids level of patients with increased risk for colorectal cancer	10	4/6	250 mg of dry ginger root extract (5% gingerols)/cap, 8 capsules/day	10	3/7	250 mg of lactose/cap, 8 capsules/day	28 days	Double-blind
Tilburg et al. (2014) [54]	United States	Randomized controlled trial	Irritable bowel syndrome	15	N/A	1 g of ginger in capsules (2.29 mg/g of gingerols and 6-shogaol)	15	N/A	Brown sugar in capsules	28 days	Double-blind
	United States	Randomized controlled trial	Irritable bowel syndrome	15	N/A	2 g of ginger in capsules (2.29 mg/g of gingerols and 6-shogaol)					
Wigler et al. (2003) [55]	Israel	Crossover randomized controlled trial	Symptomatic gonarthritis	Group 1 (ginger first): 14; Group 2 (placebo first): 15	Group 1 (ginger first): 1/13 Group 2 (placebo first): 5/10	250 mg of ginger extract (10 mg of gingerol)/cap, 4 capsules/day	Group 2 (placebo first): 15; Group 1 (ginger first): 14	Group 2 (placebo first): 5/10; Group1 (ginger first): 1/13	Placebo capsules, 4 capsules/day	Two periods of 12 weeks	Double-blind

* DMA regimen: dexamethasone, metoclopramide, and aprepitant; M/F: Male/Female; N/A: Not available.

**Table 2:** Evaluation system and key finding of the included studies with a “high quality of evidence” (quality assessment score is of at least 10).

Author (Year)	Evaluation Outcome System	Main Result	Adverse Effect
Marx et al. (2017) [17]	FLIE-5DR questionnaire, RINVR	Compared with placebo, ginger supplementation therapy can improve the chemotherapy-induced nausea-related quality of life and relieve vomiting and fatigue caused by chemotherapy.	No
Sanaati et al. (2016) [18]	Effects of the groups on nausea and vomiting using the generalized estimating equations (GEE) model	Ginger treatment reduced the frequency of vomiting and nausea significantly.	No
Thamlikitkul et al. (2016) [19]	Nausea score ( by VAS), vomiting incidence, rate of rescue medication use, and incidence of chemotherapy dose reduction	This study indicated that taking 1g of ginger for five days from the first day of chemotherapy had no effect in reducing the nausea severity of breast cancer patients receiving Adriamycin and cyclophosphamide chemotherapy.	No
Li et al. (2017) [20]	Incidence and severity of CINV by the MASCC Antiemesis Tool	In lung cancer patients who received cisplatin regimen, taking ginger as an adjuvant drug for antiemetics was ineffective in reducing the incidence and severity of CINV.	No
Ansari et al. (2016) [21]	Episodes of vomiting and nausea severity by nausea and vomiting grading	There was no significant improvement in breast cancer patients receiving chemotherapy regime-induced CINV upon ginger treatment. Thus, additional study is needed to make a conclusion.	No
Sharifzadeh et al. (2018) [4]	Rhodes questionnaire 2	In relieving moderate to mild nausea and vomiting caused by pregnancy, ginger groups had similar effects to vitamin B6 and were more effective than the placebo group.	No
Matsumura et al. (2015) [22]	Creatine kinase, lactate dehydrogenase, 1RM, muscle soreness (by VAS), Circumference, ROM-flexion, ROM-extension, skin temp—non-dominant arm, skin temp—dominant arm	This study showed that taking 4 g of ginger may promote the recovery of muscle strength after intense exercise but has no effect on indicators of muscle damage or delayed onset muscle soreness.	No
Martins et al. (2018) [23]	Four-point scale, faces pain scale, visual numeric scale, nausea, ordinal scale, photophobia, phonophobia, and treatment satisfaction	The ginger-treated group showed a significant effect in reducing migraine attacks.	No
Arzati et al. (2017) [24]	FBS, total cholesterol, TG, LDL-cholesterol, HDL-cholesterol, HbA1C	Ginger supplementation significantly lowered fasting blood sugar, the mean variation of HbA1C, and LDL/HDL ratio.	No
Attari et al. (2016) [25]	Glucose, leptin, resistin, adiponectin, insulin, HOMA-IR, QUICKI	A little beneficial effect of ginger powder supplementation was found regarding improving biochemical obesity indicator and weight loss.	No
Attari et al. (2015) [26]	Obesity-associated parameters ^1^	Subjects with the *AA*, *UCP1*, and *Trp64Trp* genotypes of β3ADR had significantly decreased anthropometric measurements and total appetite scores compared with the placebo group, but other evaluation outcomes were not significant.	No
Mozaffari-Khosravi et al. (2016) [13]	Serum TNF-α, serum IL-1β	In knee osteoarthritis patients, serum TNF-αNF-m IL-1β was decreased in both groups, with a lower level in the ginger group than the placebo group.	No
Aryaeian et al. (2019) [27]	Disease activity score-28, Gene expression levels of *PPARγ*, *FOXP3*, *T-bet*, *GATA3*, *RORγt,* and *NFκB*	This study showed that ginger could improve the active rheumatoid arthritis, as *FOXP3* gene expression significantly increased within the ginger group and between two groups, whereas *T-bet* and *RORγt* gene expression decreased significantly between the two groups.	Yes
Kashefi et al. (2015) [28]	Percentage by which the mean hemorrhage decreased (%)	Ginger treatment reduced menstrual blood loss significantly during three interventions.	No
Paritakul et al. (2016) [29]	Breast milk volume on day three, breast milk volume on day seven, and serum prolactin level	Ginger treatment significantly increased milk volume on the third day compared to the placebo group. However, no significant difference was found in the milk volume and serum prolactin levels on the seventh day between the ginger and placebo groups.	No
Ryan et al. (2012) [30]	Seven point semantic rating, 13 item symptom inventory, and functional assessment of chronic illness therapy general	In adult cancer patients, a daily dose of 0.5–1.0 g of ginger was helpful in relieving the severity of acute chemotherapy-induced nausea	Yes
Zick et al. (2008) [31]	Prevalence and severity of delayed nausea and vomiting (by Morrow Assessment of Nausea and Emesis questionnaire)	Taking ginger as a reduction of CINV was insufficient, and there was no additional benefit for reducing the severity of acute and delayed CINV.	No
Fahimi et al. (2010) [32]	Prevalence, severity, and duration of acute and delayed nausea and vomiting (by Morrow Assessment of Nausea and Emesis)	Ginger treatment showed no effect in reducing the prevalence, severity, and duration of both acute and delayed nausea and vomiting	No
Yekta et al. (2012) [33]	Self-made, two-part self-reporting instrument (number of vomiting, use of other antiemetics, side effects)	The ginger treatment group decreased vomiting at anticipatory, acute, and delayed phases of patients who received chemotherapy.	Yes
Ensiyeh et al. (2009) [3]	Nausea scores, average number of vomiting episodes, follow-up visit, a five-point Likert scale	In early pregnancy, ginger intake had a stronger effect on relieving the severity of nausea than vitamin B6 intake. However, it had no significant difference in decreasing the number of vomiting episodes.	No
Willetts et al. (2003) [34]	Rhodes index of nausea, vomiting, and retching	Regarding nausea experience and retching, the ginger group was significantly lower than the placebo group of pregnancy-induced nausea.	No
Vutyavanich et al. (2001) [35]	Nausea (VAS score), number of vomiting episodes, and symptoms assessed by Likert scales	Nausea and vomiting induced in pregnancy could be relieved by ginger.	No
Fischer-Rasmussen et al. (1990) [36]	The severity of hyperemesis (by degree of nausea, vomiting, and weight loss)	Ginger treatment showed significantly greater relief on hyperemesis than a placebo.	No
Smith et al. (2004) [37]	Incidence of nausea, dry retching, and vomiting (by Rhodes index of nausea and vomiting form) and health status (by MOS 36 short form health survey)	Pregnant women with nausea, dry retching, vomiting can use ginger in the early stage of pregnancy to relieve the severity of symptoms as effectively as vitamin B6	No
Biswas et al. (2011) [38]	The severity of dysmenorrhea, nausea, and vomiting (by VAS), average of nausea spells per day, vomiting episodes, average of nausea episodes, and average number of vomiting in last week	A ginger extract can be considered as safe therapy and effective alternative for the reduction of nausea and vomiting with no severe or serious adverse events.	No
Firouzbakht et al. (2014) [39]	The severity of nausea and the frequency of vomiting (by Likert scale and VAS)	After one week, the severity of nausea and vomiting was reduced dramatically in 60.6%, 42.7% and 61% of the ginger, placebo, and B6 groups, respectively.	Yes
Arfeen et al. (1995) [40]	The incidence of PONV and the distribution of nausea score	0.5 or 1.0 g of ginger showed no efficacy on the incidence by postoperative nausea and vomiting.	No
Eberhart et al. (2003) [41]	The incidence rate of PONV, nausea, vomiting, and rescue antiemetics	There was no reduction in nausea, vomiting and the demand for antiemetic rescue treatment in three groups.	Yes
Mandal et al. (2014) [42]	Episodes of nausea, retching, vomiting and rescue antiemetic (by the score of Bellville), and severity of PONV (by VAS)	Ginger combined with ondansetron may be more helpful in controlled PONV than ondansetron alone.	No
Ozgoli et al. (2009) [43]	Self-administered questionnaire	When alleviating pain in women with primary dysmenorrhea, ginger was comparable to mefenamic acid and ibuprofen.	No
Kashefi et al. (2013) [44]	PVAS	There were differences in pain after administration in the ginger and zinc sulfate groups, and when compared with placebo groups, it was shown to be effective in both groups.	Yes
Rahnama et al. (2012) [45]	Pain (by VAS), duration of pain	There were significant differences in the severity of pain between the two groups, but only ‘protocol 1’ showed a significant difference in the duration of pain between two groups.	No
Black et al. (2010) [46]	Elbow range of motion, arm volume, VAS score, and metabolic rate	A single 2 g dose of ginger did not attenuate eccentric exercise-induced muscle pain, inflammation or dysfunction at 45 min after ingestion. However, ginger may attenuate the day-to-day progression of muscle pain.	No
Black et al. (2009) [47]	Arm muscle pain intensity muscle soreness (mm) 24-post, range of motion, Arm volume, Isometric force, △ROM (mean percent change in range of motion, %), △Volume (percent change in arm volume, %), △Force (percent change in isometric force, %), PGE2 (pg/mL), and ratings of perceived exertion	Taking raw and heat-treated ginger helped to reduce muscle pain following exercise-induced muscle injury.	No
Mahluji et al. (2013) [48]	Serum FBG, HbA1c, insulin, HOMA, QUICKI, TG, TC, LDL-C, HDL-C	Ginger supplementation had a significant effect in reducing the levels of insulin, LDL-C, TG, and HOMA, and it increased the QUICKI index compared to the control group.	Yes
Khandouzi et al. (2013) [49]	FBS, HbA1c, ApoB, Apo A-I, ApoB/Apo A-I, MDA	Ginger supplementation may help reduce FBS, HbA1c, ApoB, Apo A-I, ApoB/Apo A-I, MDA levels, as compare to the placebo and the baseline groups.	No
Phillips et al. (1992) [50]	Paracetamol concentration (time to peak and time to the first detection)	The oral ingestion of 1 g of ginger simultaneously with paracetamol did not affect the rate of absorption of paracetamol. Therefore, the study revealed that ginger had no better effect on gastric motility.	No
Jiang et al. (2013) [14]	cyclooxygenase-1 and 15-hydroxyprostaglandin protein levels	In the high risk of CRC participants, the colonic cyclooxygenase-1 protein level significantly decreased in the ginger group. On the other hand, 15-hydroxyprostaglandin was unchanged. There is no significant difference in average risk for CRC between the ginger and placebo groups.	No
Citronberg et al. (2013) [51]	Bax, Bcl-2, p21, hTERT, MIB-1, Bax/Bcl-2 Ratio, Bax/hTERT Ratio, Bax/MIB-1 Ratio, p21/hTERT Ratio, p21/MIB-1 Ratio, cell cycle score (w/MIB-1), and cell cycle score (w/hTERT)	Two grams of ginger extract may help in reducing the proliferation of normal-appearing colorectal epithelium, as well as increased apoptosis and differentiation relative to proliferation—especially in the differentiation zone of crypts	Yes
Zick et al. (2011) [52]	PGE2, 13-hydroxy-octadecadienoic acid, and 5-, 12-, and 15 hydroxyeicosatetraenoic acid	Ginger treatment may help to reduce eicosanoid levels by inhibiting synthesis from arachidonic acid. Additionally, ginger is considered to be safe for people with a high risk of colorectal cancer.	No
Zick et al. (2014) [53]	PGE2, LTB4, 13-hydroxy-octadecadienoic acids, and 5-, 12-, and 15-hydroxy-eicosatetraenoic acid	Treating root extraction with ginger for people at high risk for CRC for 28 days significantly decreased risk in the normal colonic mucosa of arachidonic acid and significantly increased LTB4, but other eicosanoids were ineffective.	Yes
Tilburg et al. (2014) [54]	Irritable bowel syndrome severity scale and adequate relief rating scale	In treating irritable bowel syndrome, ginger may not be a proper choice because the result of study could not suggest evidence for the better performance of the ginger treatment.	Yes
Wigler et al. (2003) [55]	VAS of pain on movement and handicap	After the crossover (three months), the ginger treatment group showed a significantly higher effect compared to the placebo group.	Yes

1: Body weight, BMI, waist circumference, hip circumference, waist-to-hip ratio, waist-to-height-ratio, body fat, body fat mass, fat-free body mass, appetite total score. CINV: chemotherapy-induced nausea and vomiting; FBS: fasting blood sugar; FLIE-5DR: functional living index emesis 5-day recall; HOMA-IR: homeostasis model assessment of insulin resistance; HDL-C: high-density lipoprotein-cholesterol; HbA1c: Hemoglobin A1c; IL: interleukin; LDL-C: low-density lipoprotein-cholesterol; MDA: malondialdehyde; PVAS: pain visual analog scale; QUICKI: quantitative insulin sensitivity check index; RINVR: Rhodes inventory of nausea, vomiting and retching; ROM: range of motion; TNF-α: tumor necrosis factor-alpha; VAS: visual analog scale; MASCC: multinational association of supportive care in cancer; Apo: apolipoprotein; TC: total cholesterol; TG: triglyceride; LTB4: leukotriene B4; PG: prostaglandins treatment.

## Supplementary Materials

The following are available online at https://www.mdpi.com/2072-6643/12/1/157/s1, Table S1: PRISMA checklist, Table S2: Study design and demographic description of the included studies with a quality score less than 10, Table S3: Evaluation system and key finding of the included studies with a quality score less than 10, Table S4: Types of adverse effect, incidence rate, and dosage from 17 trials, Table S5: Quality assessment outcomes and quality assessment scores of the included studies.
