# Supplementary material for: Ginger on Human Health: A Comprehensive Systematic Review of 109 Randomized Controlled Trials
Source: Nutrients. 2020 Jan 6;12(1):157. doi: 10.3390/nu12010157 (PMC7019938; doi:10.3390/nu12010157)
Supplement: Supplementary file 1 [file nutrients-12-00157-s001.pdf]

PRISMA 2009 Checklist

Table S1. PRISMA checklist.

Section/topic	#	Checklist item	Reported on page #
**TITLE**			
Title	1	Identify the report as a systematic review, meta-analysis, or both.	1
**ABSTRACT**			
Structured summary	2	Provide a structured summary including, as applicable: background; objectives; data sources; study eligibility criteria, participants, and interventions; study appraisal and synthesis methods; results; limitations; conclusions and implications of key findings; systematic review registration number.	1
**INTRODUCTION**			
Rationale	3	Describe the rationale for the review in the context of what is already known.	1-2
Objectives	4	Provide an explicit statement of questions being addressed with reference to participants, interventions, comparisons, outcomes, and study design (PICOS).	2
**METHODS**			
Protocol and registration	5	Indicate if a review protocol exists, if and where it can be accessed (e.g., Web address), and, if available, provide registration information including registration number.	N/A
Eligibility criteria	6	Specify study characteristics (e.g., PICOS, length of follow-up) and report characteristics (e.g., years considered, language, publication status) used as criteria for eligibility, giving rationale.	2
Information sources	7	Describe all information sources (e.g., databases with dates of coverage, contact with study authors to identify additional studies) in the search and date last searched.	2
Search	8	Present full electronic search strategy for at least one database, including any limits used, such that it could be repeated.	2
Study selection	9	State the process for selecting studies (i.e., screening, eligibility, included in systematic review, and, if applicable, included in the meta-analysis).	2
Data collection process	10	Describe method of data extraction from reports (e.g., piloted forms, independently, in duplicate) and any processes for obtaining and confirming data from investigators.	3
Data items	11	List and define all variables for which data were sought (e.g., PICOS, funding sources) and any assumptions and simplifications made.	3
Risk of bias in individual studies	12	Describe methods used for assessing risk of bias of individual studies (including specification of whether this was done at the study or outcome level), and how this information is to be used in any data synthesis.	N/A
Summary measures	13	State the principal summary measures (e.g., risk ratio, difference in means).	N/A
Synthesis of results	14	Describe the methods of handling data and combining results of studies, if done, including measures of consistency (e.g., I^2) for each meta-analysis.	N/A

PRISMA 2009 Checklist

Section/topic	#	Checklist item	Reported on page #
Risk of bias across studies	15	Specify any assessment of risk of bias that may affect the cumulative evidence (e.g., publication bias, selective reporting within studies).	3
Additional analyses	16	Describe methods of additional analyses (e.g., sensitivity or subgroup analyses, meta-regression), if done, indicating which were pre-specified.	N/A
**RESULTS**			
Study selection	17	Give numbers of studies screened, assessed for eligibility, and included in the review, with reasons for exclusions at each stage, ideally with a flow diagram.	3
Study characteristics	18	For each study, present characteristics for which data were extracted (e.g., study size, PICOS, follow-up period) and provide the citations.	4-13
Risk of bias within studies	19	Present data on risk of bias of each study and, if available, any outcome level assessment (see item 12).	N/A
Results of individual studies	20	For all outcomes considered (benefits or harms), present, for each study: (a) simple summary data for each intervention group (b) effect estimates and confidence intervals, ideally with a forest plot.	14-22
Synthesis of results	21	Present results of each meta-analysis done, including confidence intervals and measures of consistency.	N/A
Risk of bias across studies	22	Present results of any assessment of risk of bias across studies (see Item 15).	22-23
Additional analysis	23	Give results of additional analyses, if done (e.g., sensitivity or subgroup analyses, meta-regression [see Item 16]).	N/A
**DISCUSSION**			
Summary of evidence	24	Summarize the main findings including the strength of evidence for each main outcome; consider their relevance to key groups (e.g., healthcare providers, users, and policy makers).	23-25
Limitations	25	Discuss limitations at study and outcome level (e.g., risk of bias), and at review-level (e.g., incomplete retrieval of identified research, reporting bias).	23-25
Conclusions	26	Provide a general interpretation of the results in the context of other evidence, and implications for future research.	25-26
**FUNDING**			
Funding	27	Describe sources of funding for the systematic review and other support (e.g., supply of data); role of funders for the systematic review.	26

From: Moher D, Liberati A, Tetzlaff J, Altman DG, The PRISMA Group (2009). Preferred Reporting Items for Systematic Reviews and Meta-Analyses: The PRISMA Statement. PLoS Med 6(7): e1000097. doi:10.1371/journal.pmed1000097

For more information, visit: **www.prisma-statement.org**.

Table S2. Study design and demographic description of the included studies with a quality score less than 10.

Author (year)	Cohort allocation	Study design	Type of disease/symptom	Intervention			Comparator			Duration	Blind
				Number	M/F	Dosage	Number	M/F	Dosage		
Bossi *et al.* (2017) [1]	Italy	Randomized controlled trial	Chemotherapy-induced nausea and vomiting	121	83/38	40 mg of ginger extract/cap, 4 capsules/day, bid	123	77/46	4 placebo capsules/day, bid	42 to 56 days	Double-blind
Konmun *et al.* (2017) [2]	Thailand	Randomized controlled trial	Chemotherapy-induced nausea and vomiting	40	4/36	5 mg of 6-gingerol (1.4% w/w of ginger extract)/cap, 4 capsules/day, bid	41	2/39	Capsule containing diluents/binder, 4 capsules/day, bid	12 weeks	Double-blind
Zeraati *et al.* (2016) [3]	Iran	Randomized controlled trial	Postoperative nausea and vomiting	46	0/46	25 drops of ginger extract in 30 mL of water	46	0/46	30 mL of water	1 h before surgery	Double-blind
Rad *et al.* (2018) [4]	Iran	Crossover randomized controlled trial	Primary dysmenorrhea	168	0/168	200 mg of powdered ginger/cap, 4 capsules/day	168	0/168	800 mg of ibuprofen/cap, 4 capsules/day	Two consecutive cycles (at the onset of pain)	Double-blind
Shirvani *et al.* (2017) [5]	Iran	Randomized controlled trial	Primary dysmenorrhea	61	0/61	250 mg of powdered ginger capsule	61	0/61	Exercise	8 weeks (at the onset of menstruation and every 6 h until pain relief)	Open-label
Mashak *et al.* (2018) [6]	Iran	Randomized controlled trial	Post-spinal puncture headache	80	0/80	250 mg of powdered ginger/cap, 3 capsules/dose	80	0/80	No intervention	Single-dose (24 h before the C-section)	Open-label
Hashemi *et al.* (2019) [7]	Iran	Randomized controlled trial	Low back pain	40	N/A	Dose N/A (Ginger)	40	N/A	Dose N/A (Control)	N/A	Single-blind
	Iran	Randomized controlled trial	Low back pain	40	N/A	Dose N/A (Ginger)	40	N/A	Dose N/A (vitamin D)	N/A	Single-blind

Hasanvand *et al.* (2018) [8]	Iran	Randomized controlled trial	Chest pain by percutaneous transluminal coronary angioplasty	17	9/8	250 mg of powdered ginger/cap, 1 capsule/day	17	13/4	250 mg of starch/cap, 1 capsule/day	7 days from 10 days before angioplasty	Double-blind
Azimi *et al.* (2016) [9]	Iran	Randomized controlled trial	Type 2 diabetes mellitus	41	15/26	3 g of powdered ginger + black tea, three times a day	40	16/24	3 g of cinnamon + black tea, three times a day	8 weeks	Single-blind
	Iran	Randomized controlled trial	Type 2 diabetes mellitus	41	15/26	3 g of powdered ginger + black tea, three times a day	42	17/25	3 g of cardamom + black tea, three times a day	8 weeks	Single-blind
	Iran	Randomized controlled trial	Type 2 diabetes mellitus	41	15/26	3 g of powdered ginger + black tea, three times a day	42	16/26	1 g of saffron + black tea, three times a day	8 weeks	Single-blind
	Iran	Randomized controlled trial	Type 2 diabetes mellitus	41	15/26	3 g of powdered ginger + black tea, three times a day	39	15/24	Black tea, three times a day	8 weeks	Single-blind
Shidfar *et al.* (2015) [10]	Iran	Randomized controlled trial	Type 2 diabetes mellitus	22	N/A	1 g of powdered ginger/cap, 3 capsules/day	23	N/A	1 g of lactose/cap, 3 capsules/day	3 months	Double-blind
Tabibi *et al.* (2015) [11]	Iran	Randomized controlled trial	Serum lipids and lipoproteins levels in peritoneal dialysis patients	18	11/7	250 mg of ginger/cap, 4 capsules/day	18	10/8	250 mg of starch/cap, 4 capsules/day	10 weeks	Double-blind
Karimi *et al.* (2015) [12]	Iran	Randomized controlled trial	Metabolic syndrome and pro-inflammatory markers of obese women who have diagnosed with breast neoplasms	10	0/10	750 mg of powdered ginger/cap, 4 capsules/day	10	0/10	1 g of starch/cap, 4 capsules/day	7 days a week and for 6 weeks.	N/A
	Iran	Randomized controlled trial	Metabolic syndrome and pro-inflammatory	10	0/10	750 mg of powdered ginger/cap, 4	10	0/10	Exercise	6 weeks and 4 sessions at a week	N/A

			markers of obese women who have diagnosed with breast neoplasms			capsules/day + exercise					
Emrani *et al.* (2016) [13]	Iran	Randomized controlled trial	Antituberculosis-induced gastrointestinal adverse reactions	30	22/8	250 mg of powdered ginger/cap, 2 capsules/day	30	25/5	2 placebo capsules/day	4 weeks (at 30 min before morning antituberculosis medications)	Double-blind
Kulkarni *et al.* (2016) [14]	India	Randomized controlled trial	Anti-inflammatory and antioxidant effect in tuberculosis	34	N/A	250 mg of ginger extract/cap (1.5 g pure powder), 2 capsules/day	35	N/A	250mg of starch/cap, 2 capsules/day	1 month	N/A
Miyamoto *et al.* (2015) [15]	Japan	Crossover randomized controlled trial	Thermoregulatory function and fat oxidation: thermal balance, T_{cor}[1], and blood energy substrates levels	5	5/0	250 mg of dried ginger root powder/cap, 4 capsules/dose	5	5/0	250 mg of starch/cap, 4 capsules/dose	Single dose	N/A
	Japan	Crossover randomized controlled trial	Thermoregulatory function and fat oxidation: threshold T_{cor}[1] for skBF[2] and m_{sw}[3]	4	4/0	250 mg of dried ginger root powder/cap, 4 capsules/dose	4	4/0	250 mg of starch/cap, 4 capsules/dose	Single dose	N/A
Manusirivithaya *et al.* (2004) [16]	Thailand	Crossover randomized controlled trial	Chemotherapy-induced nausea and vomiting	43	N/A	1 g of powdered ginger root/day	43	N/A	Placebo (acute) and metoclopramide (delayed)	5 days per chemotherapy cycle[4]	Double-blind
Pillai *et al.* (2011) [17]	India	Randomized controlled trial	Chemotherapy-induced nausea and vomiting	27	N/A	1000 mg or 2000 mg of powdered ginger/day[5]	30	16/14	1000 mg and 2000 mg of starch powder/day	Day 1 to day 3 of chemotherapy cycle	Double-blind

Panahi *et al.* (2012) [18]	Iran	Randomized open-label controlled trial	Chemotherapy-induced nausea and vomiting	37	0/37	500 mg of powdered ginger and standard antiemetic regimen[6]	41	0/41	Standard antiemetic regimen	4 days from initiation of chemotherapy	Open-label
Sontakke *et al.* (2002) [19]	India	Crossover randomized controlled trial	Chemotherapy-induced nausea and vomiting	50	11/39	500 mg of powdered ginger/cap, 2 ginger capsules + 2 mL of normal saline (i.v.) 20 min before chemotherapy + 2 ginger capsules after 6 h	50	11/39	2 lactulose capsules + metoclopramide 20 mg (i.v.) 20 min before chemotherapy + 2 capsules of 5 mg of metoclopramide/cap	3 cycle (21 days of interval between cycles)	Double-blind
	India	Crossover randomized controlled trial	Chemotherapy-induced nausea and vomiting	50	11/39	500 mg of powdered ginger/cap, 2 ginger capsules + 2 mL of normal saline (i.v.) 20 min before chemotherapy + 2 ginger capsules after 6 h	50	11/39	2 lactulose capsules + ondansetron 4 mg (i.v.) 20 min before chemotherapy + 2 capsules of 2 mg of ondansetron/cap	3 cycle (21 days of interval between cycles)	Double-blind
Arslan *et al.* (2014) [20]	Turkey	Randomized controlled trial	Chemotherapy-induced nausea and vomiting	30	30/0	500 mg of powdered ginger mixed in yogurt, twice/day	30	30/0	No intervention	3 days	Open label
Mohammad beigi *et al.* (2011) [21]	Iraq	Randomized controlled trial	Nausea and vomiting of pregnancy	34	0/34	200 mg of ginger essence/cap, 3 capsules/day	34	0/34	10 mg of metoclopramide/cap , 3 capsules/day	5 days	Double-blind
	Iraq	Randomized controlled trial	Nausea and vomiting of pregnancy	34	0/34	200 mg of ginger essence/cap, 3 capsules/day	34	0/34	200 mg of flour/cap, 3 capsules/day	5 days	Double-blind
Ozgoli *et al.* (2009) [22]	Iran	Randomized controlled trial	Nausea and vomiting of pregnancy	32	0/32	250 mg of powdered ginger root/cap, 4 capsules/day	35	0/35	250 mg of lactose/cap, 4 capsules/day	4 days	Single-blind

Keating *et al.* (2002) [23]	United States	Randomized controlled trial	Nausea and vomiting of pregnancy	13	0/13	1 tablespoon of commercially prepared study syrup (250 mg of ginger with honey, water), 4 times daily	11	0/11	1 tablespoon of commercially prepared placebo syrup (honey, lemon oil, water), 4 times daily	2 weeks	Double-blind
Pongrojpaw *et al.* (2007) [24]	Thailand	Randomized controlled trial	Nausea and vomiting of pregnancy	85	0/85	500 mg of powdered ginger/cap, 2 capsules/day, bid	85	0/85	50 mg of dimenhydrinate/cap, 2 capsules/day, bid	1 week	Double-blind
Saberi *et al.* (2013) [25]	Iran	Randomized controlled trial	Nausea and vomiting of pregnancy	37	0/37	250 mg of powdered ginger/cap, 3 capsules/day	36	0/36	3 lactose capsules/day (Placebo)	4 days	N/A
	Iran	Randomized controlled trial	Nausea and vomiting of pregnancy	37	0/37	250 mg of powdered ginger/cap, 3 capsules/day	33	0/33	No intervention (Control)	4 days	N/A
Javadi *et al.* (2013) [26]	Iran	Randomized controlled trial	Nausea and vomiting of pregnancy	47	0/47	250 mg of powdered ginger/cap, 4 capsules/day	48	0/48	40 mg of vitamin B6/tab, 2 tablets/day	4 days	N/A
Apariman *et al.* (2006) [27]	Thailand	Randomized controlled trial	Postoperative nausea and vomiting	30	N/A	500 mg of powdered ginger/cap, 3 capsules/day	30	N/A	3 capsules of placebo/day	1 h prior to the operation	N/A
Nanthakomon *et al.* (2006) [28]	Thailand	Randomized controlled trial	Postoperative nausea and vomiting	60	N/A	500 mg of powdered ginger/cap, 2 capsules/dose	60	N/A	500 mg of lactose/cap, 2 capsules/dose	Single-dose (one hour before the surgery)	Double-blind
Kalava *et al.* (2013) [29]	United States	Randomized controlled trial	Postoperative nausea and vomiting	116	0/116	1 g of powdered ginger/cap, 1 capsule/dose	123	0/123	1 g of placebo/cap, 1 capsule/dose	30 min before induction of anesthesia and 2 hours after surgery	Double-blind
Visalyaputra *et al.* (1997) [30]	Thailand	Randomized controlled trial	Postoperative nausea and vomiting	Group 3 (ginger): 27	0/27	500 mg of ginger root/cap, 2 capsules/dose + 0.5	Group 1 (placebo): 28	0/28	500 mg of dried rice starch/cap, 2 capsules/dose + 0.5	1 h before induction of anesthesia and	Triple-blind

						mL of normal saline (i.v.)			mL of normal saline (i.v.)	30 min before discharge	
	Thailand	Randomized controlled trial	Postoperative nausea and vomiting	Group 4 (droperidol + ginger): 27	0/27	500 mg of ginger root/cap, 2 capsules/dose + 1.25 mg of droperidol in 0.5 mL of normal saline (i.v.)	Group 2 (droperidol): 29	0/29	500 mg of rice starch/cap, 2 capsules/dose + 1.25 mg of droperidol in 0.5 mL of normal saline (i.v.)	1 h before induction of anesthesia and 30 min before discharge	Triple-blind
Phillips *et al.* (1993) [31]	United Kingdom	Randomized controlled trial	Postoperative nausea and vomiting	40	0/40	1 g of powdered ginger root in 2 capsules	40	0/40	5 mg of metoclopramide/cap, 2 capsules/dose	Single-dose	Double-blind
	United Kingdom	Randomized controlled trial	Postoperative nausea and vomiting	40	0/40	1 g of powdered ginger root in 2 capsules	40	0/40	1 g of lactose in 2 capsules	Single-dose	Double-blind
Bone *et al.* (1990) [32]	United Kingdom	Randomized controlled trial	Postoperative nausea and vomiting	20	0/20	500 mg of powdered ginger root/cap and injection of 2 mL of sterile water	20	0/20	500 mg of lactulose/cap and injection of 2 mL of sterile water	Single-dose	Double-blind
	United Kingdom	Randomized controlled trial	Postoperative nausea and vomiting	20	0/20	500 mg of powdered ginger root/cap and injection of 2 mL of sterile water	20	0/20	500 mg of lactulose/cap and injection of 10 mg of metoclopramide	Single-dose	Double-blind
Tavlan *et al.* (2006) [33]	Turkey	Randomized controlled trial	Postoperative nausea and vomiting	60	6/54	Dexamethasone 150 μg/kg (i.v.) + 0.5 g of ginger + 10 mg of oral diazepam	60	8/52	Dexamethasone 150 μg/kg (i.v.) + placebo + 10 mg of oral diazepam	Dexamethasone: before the induction of anesthesia Ginger, placebo and diazepam: 1 h prior to surgery	Double-blind
Dabaghzadeh *et al.* (2014) [34]	Iran	Randomized controlled trial	Antiretroviral-induced nausea and vomiting	51	36/15	250 mg of powdered ginger/cap, 4 capsules/day, bid	51	34/17	250 mg of starch/cap, 4 capsules/day, bid	14 days	Double-blind

Holtmann *et al.* (1989) [35]	Germany	Randomized controlled trial	Emesis	38	18/20	One capsule containing 1000 mg of powdered ginger root	38	18/20	One capsule containing 100 mg of dimenhydrinate	N/A	Double-blind
	Germany	Randomized controlled trial	Emesis	38	18/20	One capsule containing 1000 mg of powdered ginger root	38	18/20	One capsule containing lactose	N/A	Double-blind
Grontved *et al.* (1988) [36]	Denmark	Randomized controlled trial	Seasickness	40	N/A	1 g of powdered ginger root	39	N/A	1 g of lactose	Single-dose	Double-blind
Grontved *et al.* (1985) [37]	Denmark	Crossover randomized controlled trial	Vertigo and nystagmus	8	N/A	1 g of powdered ginger root/cap, 1 capsule/dose	8	N/A	1 g of lactose/cap, 1 capsule/dose	Single-dose	Double-blind
Mowrey *et al.* (1982) [38]	United States	Randomized controlled trial	Motion sickness	12	6/6	2 capsules of 940 mg of powdered ginger	Group 1: 12<br>Group 2: 12	Group 1: 6/6<br>Group 2: 6/6	Group 1: 100 mg of dimenhydrinate<br>Group 2: 2 capsules of powdered chickweed herb (placebo)	Single-dose	N/A
Stewart *et al.* (1990) [39]	United States	Randomized controlled trial	Motion sickness	Group 1: 8<br>Group 2: 8<br>Group 3: 8	Group 1: 8/0<br>Group 2: 8/0<br>Group 3: 8/0	Group 1: 500 mg of powdered ginger<br>Group 2: 1000 mg of powdered ginger<br>Group 3: 1000 mg of fresh ginger	Scopolamine group: 8<br>Control group: 8	Scopolamine group: 8/0<br>Control group: 8/0	Scopolamine group: 0.6 mg of scopolamine<br>Control group: lactose	1 h before the test (ginger and lactose) and 30 min before the test (scopolamine)	N/A
Jenabi *et al.* (2013) [40]	Iran	Randomized controlled trial	Primary dysmenorrhea	35	0/35	500 mg of powdered ginger/cap, 3 capsules/day	34	0/34	3 capsules of placebo/day	First menstruation cycles for 3 days	N/A
Shirvani *et al.* (2014) [41]	Finland	Randomized controlled trial	Primary dysmenorrhea	61	0/61	250 mg of powdered ginger/cap, 4 capsules/day	61	0/61	250 mg of mefenamic acid/cap, 3 capsules/day	2 cycles of menstruation (from the onset	N/A

										of menstruation until pain relief)	
Black *et al.* (2008) [42]	United States	Crossover randomized controlled trial	Quadriceps muscle pain during moderate-intensity cycling exercise	25	10/15	Six capsules containing 2 g of ground ginger with 250 mL of water and 1 tablespoon of olive oil	25	10/15	Six capsules containing 2 g of placebo with 250 mL of water and 1 tablespoon of olive oil	Single-dose	Double-blind
Maghbooli *et al.* (2014) [43]	Iran	Randomized controlled trial	Migraine	50	13/37	250 mg of powdered ginger rhizome/cap, 1 capsule/onset of migraine	50	16/34	50 mg powder of sumatriptan/cap, 1 capsule/onset of migraine	Single-dose	Double-blind
Arablou *et al.* (2013) [44]	Iran	Randomized controlled trial	Type 2 diabetes mellitus	33	25/8	800 mg of powdered ginger rhizome/cap, 2 capsules/day	30	23/7	800 mg of wheat flour/cap, 2 capsules/day	12 weeks	Double-blind
Mozaffari-Khosravi *et al.* (2014) [45]	Iran	Randomized controlled trial	Type 2 diabetes mellitus	41	18/23	1 g of powdered ginger/cap, 3 capsules/day	40	13/27	1 g of cellulose microcrystalline/cap, 3 capsules/day	8 weeks	Double-blind
Andallu *et al.* (2001) [46]	India	Randomized controlled trial	Type 2 diabetes mellitus	8	8/0	500 mg of powdered ginger/cap, 2 capsules/day	8	8/0	N/A (Control group)	30 days	N/A
	India	Randomized controlled trial	Hyperglycemia and hyperlipidemia	8	8/0	500 mg of powdered ginger/cap, 2 capsules/day	Group 1: 8<br>Group 2: 8	Group 1: 8/0<br>Group 2: 8/0	Group 1: 500 mg of Ashwagandha/cap, 2 capsules/day<br>Group 2: 500 mg of Mulberry/cap, 2 capsules/day	30 days	N/A
Karimi *et al.* (2013) [47]	Iran	Randomized controlled trial	Obesity with breast cancer	10	0/10	750 mg of powdered ginger rhizome/cap, 12 capsules/day, qid	10	0/10	1 g of starch powder/cap, 12 capsules/day, qid	6 weeks	Double-blind

	Iran	Randomized controlled trial	Obesity with breast cancer	10	0/10	750 mg of powdered ginger rhizome/cap, 12 capsules/day, qid +Water-based exercise program	10	0/10	1 g of starch powder/cap, 12 capsules/day, qid + Water-based exercise program	6 weeks	Double-blind
Atashak *et al.* (2011) [48]	Iran	Randomized controlled trial	Obesity-related cardiovascular risk	8	8/0	250 mg of powdered ginger/cap, 4 capsules/day	8	8/0	250 mg of maltodextrin/cap, 4 capsules/day	10 weeks	Double-blind
	Iran	Randomized controlled trial	Obesity-related cardiovascular risk	8	8/0	250 mg of powdered ginger/cap, 4 capsules/day with resistance training	8	8/0	250 mg of maltodextrin/cap, 4 capsules/day with resistance training	10 weeks	Double-blind
Alizadeh-Navaei *et al.* (2008) [49]	Iran	Randomized controlled trial	Hyperlipidemia	45	16/29	500 mg of powdered ginger rhizome/cap, 6 capsules/day, tid	40	18/22	500 mg of lactose/cap, 6 capsules/day, tid	45 days	Double-blind
	Iran	Randomized controlled trial	Metabolic syndrome and pro-inflammatory markers of obese women who have diagnosed with breast neoplasms	10	0/10	750 mg of powdered ginger/cap, 4 capsules/day + exercise	10	0/10	Exercise	6 weeks and 4 sessions at a week	N/A
Imani *et al.* (2014) [50]	Iran	Randomized controlled trial	Serum glucose, advanced glycation end products, and inflammation in peritoneal dialysis patients	18	11/7	250 mg of ginger/cap, 4 capsules/day	18	10/8	250 mg of starch/cap, 4 capsules/day	10 weeks	Double-blind
Hu *et al.* (2011) [51]	Taiwan	Randomized controlled trial	Functional dyspepsia	N/A	N/A	400 mg of powdered ginger root/cap, 3 capsules/day	N/A	N/A	Placebo (starch)	Two afternoons, separated by at least 7 days	Double-blind

Wu *et al.* (2007) [52]	Taiwan	Randomized controlled trial	Gastric emptying and motility in healthy human	12	12/0	400 mg of powdered ginger root/cap, 3 capsules/day	12	12/0	400 mg of starch/cap, 3 capsules/day	Two afternoons, separated by at least 7 days	Double-blind
Lien *et al.* (2002) [53]	United States	Crossover randomized controlled trial	Motion sickness and gastric slow-wave dysrhythmias induced by circular vection	13	N/A	1 or 2 g of ginger in capsules	13	N/A	Placebo (starch) in capsules	Single-dose at 1 hour before circular vection	Double-blind
	United States	Crossover randomized controlled trial	Vasopressin-evoked gastric dysrhythmias and nausea	4	N/A	2 g of ginger in capsule before the infusion of vasopressin at 0.1 and 0.2 U/min iv for 30 min	4	N/A	The infusion of vasopressin at 0.1 and 0.2 U/min IV for 30 min	Two separate days, at least 3 days apart, 1 hour before the basal EGG recording	Double-blind
Shariatpanahi *et al.* (2009) [54]	Iran	Randomized controlled trial	Delayed gastric emptying, developing ventilator-associated pneumonia, clinical outcomes in adult respiratory distress syndrome	16	8/8	120 mg of ginger extract added to tube feeding/day, tid	16	8/8	1 g of coconut oil added to tube feeding/day, tid	21 days	Double-blind (nurse unblinded)
Gonlachanvit *et al.* (2003) [55]	United States	Randomized controlled trial	Hyperglycemia-evoked gastric dysrhythmias	Hyperglycemic clamping studies: 14	N/A	Hyperglycemic clamping studies: 500 mg of powdered ginger root/cap, 2 capsules/dose + 20% dextrose (i.v.)[7]	hyperglycemic clamping studies: 14	N/A	Hyperglycemic clamping studies: 2 placebo capsules/dose + 20% dextrose (i.v.)[7]	Single-dose	Double-blind

	United States	Randomized controlled trial	Hyperglycemia-evoked gastric dysrhythmias	Prostaglandin E1 (misoprostol)studies: 11	N/A	Prostaglandin E1 (misoprostol) studies: 500 mg of powdered ginger root/cap, 2 capsules/dose + 400 μg of misoprostol	prostaglandin E1 (misoprostol)studies: 11	N/A	Prostaglandin E1 (misoprostol) studies: 2 placebo capsules/dose + 400 μg of misoprostol	Single dose	Double-blind
Haghighi *et al.* (2005) [56]	Iran	Randomized controlled trial	Osteoarthritis	40	29/11	15 mg of ginger extract/cap, 2 capsules/day	40	28/12	15 mg of lactose/cap, 2 capsules/day	1 month	Double-blind
	Iran	Randomized controlled trial	Osteoarthritis	40	29/11	15 mg of ginger extract/tab, 2 tablets/day	40	32/8	400 mg of ibuprofen/cap, 3 capsules/day	1 month	Double-blind
Paramdeep *et al.* (2013) [57]	India	Randomized open-label controlled trial	Osteoarthritis of knee	Group 2: 20 Group 3: 20	Group 2: 8/12 Group 3: 6/14	Group 2: A capsule of 750 mg of powdered ginger and a capsule of placebo, twice/day Group 3: A capsule of 750 mg powdered ginger and a tablet of 50 mg of diclofenac, twice/day	Group 1: 20	Group 1: 6/14	Group 1: A capsule of placebo and a tablet of 50 mg of diclofenac, twice/day	12 weeks	Open-label
Drozdov *et al.* (2012) [58]	Russia	Randomized controlled trial	Osteoarthritis of the knee or hips	21	4/17	100 mg of ginger extract[8] + 500 mg of glucosamine/cap, 2 capsules/day	22	4/18	100 mg of diclofenac/tab, 1 tablet/day with 1000 mg of glucosamine	4 weeks	N/A
Bliddal *et al.* (2000) [59]	Denmark	Crossover randomized controlled trial	Clinical dysfunction and pain due to osteoarthritis	56	15/41	170 mg of ginger extract	56	15/41	Placebo	3 weeks	Double-blind
	Denmark	Crossover randomized controlled trial	Clinical dysfunction and pain due to osteoarthritis	56	15/41	170 mg of ginger extract	56	15/41	400 mg of ibuprofen	3 weeks	Double-blind

Gregersen *et al.* (2013) [60]	Denmark	Crossover randomized controlled trial	Diet-induced thermogenesis	22	22/0	20 g of fine-chopped ginger in the stewed apples	22	22/0	Placebo and other pungent spices[9]	Single-dose	Single-blind
Mansour *et al.* (2012) [61]	United States	Crossover randomized controlled trial	Energy expenditure, feelings of appetite and satiety and metabolic risk factors in overweight men	10	10/0	Breakfast meal with 2 g of powdered ginger dissolved in hot water beverage	10	10/0	Breakfast meal with hot beverage without ginger	Single-dose	Open-label
Janssen *et al.* (1996) [62]	Netherlands	Crossover randomized controlled trial	Anti-thrombotic effect	18	9/9	125 g of vanilla custard containing 15 g of Brazilian ginger root	18	9/9	125 g of vanilla custard	14 days	Double-blind
	Netherlands	Crossover randomized controlled trial	Anti-thrombotic effect	18	9/9	125 g of vanilla custard containing 40 g of cooked stem ginger	18	9/9	125 g of vanilla custard	14 days	Double-blind
Bordia *et al.* (1996) [63]	N/A	Randomized controlled trial	Blood lipids, blood sugar, and platelet aggregation profiling	10	N/A	4 g of powdered ginger/day for 3 months and 10 g of powdered ginger as a single dose	10	N/A	Placebo	3 months	N/A
Jiang *et al.* (2004) [64]	Australia	Crossover randomized controlled trial	Pharmacokinetics and pharmacodynamics of warfarin in healthy subjects	12	12/0	400 mg of powdered ginger rhizome/tab, 3 tablets/day	12	12/0	No intervention	1 week after warfarin administration (25 mg)	Open-label
Shariatpanahi *et al.* (2013) [65]	Iran	Randomized controlled trial	Acute respiratory distress syndrome	16	8/8	120 mg of ginger extract	16	8/8	1 g of coconut oil	21 days	Double-blind

Rouhi *et al.* (2006) [66]	Iran	Randomized controlled trial	Asthma	46	N/A	20 drops of ginger solution/8 hours[10]	46	N/A	Placebo	2 months	N/A

1: Core body temperature
2: Finger skin blood flow
3: Sweating rates
4: Two capsules of ginger (placebo) at 30 min before and one capsule at 6 and 12 h after chemotherapy together with standard antiemetics in day 1. From day 2 to day 5, they received on capsule of ginger (or metoclopramide) four times a day.
5: Patient ≥ 20 kg and < 40 kg: 6 capsules/day containing 167 mg ginger powder (total dose: 1,000 mg/day); patient ≥ 40 kg and < 60 kg: 5 capsules/day containing 400 mg ginger powder (total dose: 2,000 mg/day)
6: Granisetron plus dexamethasone
7: To achieve a plasma glucose concentration between 250 and 290 mg/dL
8: Corresponds to 2000 mg of dry ginger rhizome
9: 8.3 g of shredded horseradish, 21 g of Dijon mustard, 1.3 g of black pepper
10: 150 mg as powdered ginger in 25 drops of solution

Table S3. Evaluation system and key finding of the included studies with a quality score less than 10

Author (year)	Evaluation outcome system	Main result	Adverse effect
Bossi *et al.* (2017) [1]	Incidence and intensity of nausea (VAS), impact of nausea on daily life (FLIE questionnaire), nausea and vomiting domain scores, impact of fatigue (BFI questionnaire)	Cancer patients receiving highly emetogenic chemotherapy did not show beneficial effect of ginger treatment regarding the reduction of CINV (delayed, intercycle, and anticipatory).	No
Konmun *et al.* (2017) [2]	Complete response, intensity of nausea and appetite (by Intensity of nausea and appetite), quality of life (by Functional Assessment of Cancer Therapy-General)	In addition to highly emetogenic chemotherapy using ondansetron, metoclopramide, dexamethasone, 6-gingerol has reduced complete response rate significantly in both acute and delayed CINV.	No
Zeraati *et al.* (2016) [3]	Severity of nausea (by VAS), retching incidents	Ginger extract showed improvement in terms of reducing the incidence and mean severity score of nausea and vomiting during the cesarean section.	No
Rad *et al.* (2018) [4]	PVAS, multidimensional verbal rating scale (MVRS), and pictorial, blood loss assessment chart (PBAC)	Ginger is as effective as mefenamic acid in relieving pain with primary dysmenorrhea.	No
Shirvani *et al.* (2017) [5]	Pain intensity (by VAS), pain duration, and menstruation characteristics (menstrual cycle duration, duration on menstruation, severity of dysmenorrhea, bleeding)	Instead of taking ginger for reducing pain and dysmenorrhea, stretching exercise is safer, low-cost treatment to effectively relieve the pain.	No
Mashak *et al.* (2018) [6]	Mean arterial pressure, heart rate, arterial oxygen saturation, cesarean duration, VAS	There is significant difference in mean score of post-spinal puncture headache between ginger treatment and control group except 12 hours after C-section.	No
Hashemi *et al.* (2019) [7]	Pain (by VAS), IL-6, CRP	There is no significant difference in levels of VAS and IL-6 when comparing ginger group and vitamin B6 group, however, level of CRP in ginger group significantly decreased.	No
Hasanvand *et al.* (2018) [8]	Serum troponin I level, chest pain (by numeric rating scale)	Chest pain occurred during coronary angioplasty could be reduced by taking ginger. Also, as taking ginger is better regarding convenience and no significant complication, it could be a better choice for therapeutic agents in reducing chest pain during angioplasty.	No
Azimi *et al.* (2016) [9]	sICAM-1, systolic blood pressure, diastolic blood pressure, anthropometric measures	In within-group comparison, ginger intake significantly reduced the concentration of sICAM-1 and affected systolic blood pressure.	No
Shidfar *et al.* (2015) [10]	Serum concentration of glucose, insulin, hs-CRP, paraoxonase-1, TAC, MDA, HbA1c, HOMA-IR	Serum glucose, HbA1c, insulin, insulin resistance, hs-CRP significantly decreased while paraoxonase-1, TAC and MDA significantly increased by supplementation of ginger in T2DM patients.	No
Tabibi *et al.* (2015) [11]	Triglyceride, total cholesterol, LDL-C, HDL-C, lipoprotein (a), IL-6	Administrating 1000mg ginger daily reduces serum triglyceride concentration in peritoneal dialysis patients.	No

Karimi *et al.* (2015) [12]	IL-10, hs-CRP, insulin, insulin resistance, glucose, TG, TC, LDL-Cholesterol, HDL- Cholesterol, HDL/LDL-Cholesterol	The ginger treatment and the water-base exercise reduced the hs-CRP, IL-10, insulin, glucose, insulin resistance, LDL- Cholesterol, TG levels but increased the HDL- Cholesterol and HDL-Cholesterol /LDL- Cholesterol. The combination showed a better effect than alone supplementation.	No
Emrani *et al.* (2016) [13]	Nausea (VAS score), number of vomiting, anti-TB-induced hepatotoxicity, time to onset of anti-TB-induced hepatotoxicity	Ginger can be considered as a potential additional therapy to prevent gastrointestinal adverse reactions including hepatotoxicity, which was induced by antituberculosis.	Yes
Kulkarni *et al.* (2016) [14]	TNF-alpha, ferritin, MDA	Ginger supplementation significantly reduced the levels of TNF alpha, ferritin and MDA in ginger supplemented group in comparison to baseline. Ginger supplementation with antitubercular treatment significantly lowered TNF alpha, ferritin and MDA concentrations in comparison to control group.	No
Miyamoto *et al.* (2015) [15]	Thermal sensation and thermal comfort, oxygen consumption, CO2 production, respiratory exchange ratio, serum-free fatty acid	The effect of treating ginger seems to has no effect in peripheral and central thermoregulatory function. However, treating ginger can affect fat utilization and the treating time also can affect fat utilization.	No
Manusirivithaya *et al.* (2004) [16]	Nausea score, number of days of nausea	The addition of ginger to standard antiemetic regimen has no advantage in reducing nausea or vomiting in acute phase of cisplatin-induced emesis. In delayed phase, ginger and metoclopramide have no statistically significant difference in efficacy.	No
Pillai *et al.* (2011) [17]	ESAS	Ginger root powder was effective in reducing severity of acute and delayed CINV as additional therapy to ondansetron and dexamethasone in patients receiving high emetogenic chemotherapy.	No
Panahi *et al.* (2012) [18]	The modified form of RINVR	Standard antiemetic therapy with ginger significantly reduced prevalence of nausea 6 to 24 h post-chemotherapy. However, there is no other advantage in reducing prevalence or severity of CINV.	No
Sontakke *et al.* (2002) [19]	Control of nausea and vomiting	Efficacy of ginger in term of antiemetic was as effective as metoclopramide in cancer patients who used lose dose cyclophosphamide with cytotoxics causing mild emesis	No
Arslan *et al.* (2014) [20]	Nausea intensity, nausea, vomiting and, retching episodes	In the first 3 days of chemotherapy, administering ginger powder could be effective in reducing the severity of CINV in breast cancer patients who received anthracycline-based chemotherapy.	No
Mohammadbeigi *et al.* (2011) [21]	Vomiting, Nausea, Rhodes index	Ginger and metoclopramide groups had no significant effect, however, there was a significant difference in reducing vomiting and nausea caused by pregnancy compared to placebo group.	No
Ozgoli *et al.* (2009) [22]	Nausea intensity and vomiting episodes	Ginger treatment significantly decreased nausea intensity and vomiting episodes in pregnancy.	No
Keating *et al.* (2002) [23]	Degree of nausea and vomiting, number of vomiting episodes, daily functioning related to symptoms.	It was good option for early pregnancy to take 1g of ginger in syrup or capsules daily as antiemetics.	Yes

Pongrojpaw *et al.* (2007) [24]	Visual analogue nausea scores (VANS) and vomiting times	Ginger showed similar effect with dimenhydrinate 3-7 days after the treatment. Therefore, taking ginger can be an alternative choice for the treatment of nausea and vomiting for pregnant women.	Yes
Saberi *et al.* (2009) [25]	Nausea, vomiting, and retching (by Rhodes Index Score)	At less than 16 weeks gestation, ginger was an effective treatment for the relieving in mild to moderate nausea and vomiting in pregnant women.	No
Javadi et al. (2013) [26]	Nausea and vomiting (by MPUQE score)	There was no significant difference in efficacy of ginger and Vitamin B6 regarding reduction of symptoms of pregnancy-induced nausea.	No
Apariman *et al.* (2006) [27]	VAS, presences of vomiting at 0-2/2-6 hour postoperation	VAS in ginger group was significantly lower than placebo group at 6 hour postoperation, but there were no statistically significant differences in any other indexes.	No
Nanthakomon *et al.* (2006) [28]	Frequency of vomiting, nausea (by VAS)	Ginger treatment showed a significant effect in preventing nausea and vomiting of post major gynecologic surgery	No
Kalava *et al.* (2013) [28]	The intraoperative incidence of nausea, the number of episodes of intraoperative nausea, the incidence of intraoperative vomiting, the number of episodes of vomiting in the first 24 h after surgery, severity of nausea postoperatively	The frequency of episodes of intraoperative nausea was significantly reduced in ginger group comparing with placebo group. However, frequency of nausea, vomiting, and pain during and after an elective cesarean section was not affected by ginger.	Yes
Visalyaputra *et al.* (1997) [30]	Incidence and severity of PONV (nausea score and vomiting frequency)	Incidence of PONV after gynecological laparoscopy was not reduced by taking 2 g of ginger, 1.25 mg of droperidol or both.	No
Phillips *et al.* (1993) [31]	Nausea (number of complaints), vomiting (number of episodes), postoperative analgesic	Ginger was useful as a promising antiemetic, especially for day case surgery.	No
Bone *et al.* (1990) [32]	Incidences of nausea, vomiting, retching, sedation, abnormal movements, pain, itching and eye disturbances at the four assessment times, nausea and pain grade, postoperative use of analgesia	The antiemetics effectiveness of ginger group was significantly better than placebo group, and other antiemetic groups in patients after receiving the major gynecological surgery.	No
Tavlan *et al.* (2006) [33]	Nausea (mild, moderate, severe), retching, episode of vomiting, rescue antiemetics	Ginger treatment combination with dexamethasone for reducing postoperative nausea and vomiting did not have significant result compared with the result when using dexamethasone alone.	No
Dabaghzadeh *et al.* (2014) [34]	Nausea (by VAS), number of vomiting episodes	Ginger was useful to prevent nausea and vomiting-induced by antiretroviral regimens.	No
Holtmann *et al.* (1989) [34]	Optokinetic nystagmus, caloric response, postrotatory nystagmus	Ginger had little effect on the vestibular or the oculomotor system.	No
Grontved *et al.* (1988) [36]	Seasickness (nausea, vertigo, vomiting, and cold sweating scores)	Ginger is effective in reducing vomiting and cold sweating when compared with placebo.	No
Grontved *et al.* (1985) [37]	Vertigo score, nystagmus duration, maximum slow phase velocity of nystagmus	Motion sickness symptoms such as vertigo, nausea, vomiting, cold sweat are reduced better by taking ginger root than placebo group.	No

Mowrey *et al.* (1982) [38]	Times in revolving chair and power functions for magnitude estimations	Ginger treatment showed higher effect in reducing the gastrointestinal distress in motion sickness compared to demenhydrinate and placebo treatment.	No
Stewart *et al.* (1990) [39]	Head movements, gastric emptying, electrogastrography	Ginger did not have anti-motion sickness activity and had no function of altering gastric function during motion sickness.	Yes
Jenabi (2013) [40]	Pain (by VAS)	Ginger treatment significantly reduced VAS of post-therapy pain compared with placebo group.	No
Shirvani *et al.* (2014) [41]	Severity of dysmenorrhea 100mm visual analog scale (by VAS), severity of dysmenorrhea, pain duration, cycle duration and bleeding volume	There was no significant difference in terms of pain intensity, severity of dysmenorrhea, pain duration, cycle duration and bleeding volume between two groups. The menstrual days were longer in ginger group in first and second cycle.	No
Black *et al.* (2008) [42]	Heart rate, oxygen consumption, work rate, quadriceps muscle pain, ratings of perceived exertion, values for total mood disturbance	Compared with placebo, ginger had no clinically meaningful or statistically significant effect on perceptions of muscle pain, ratings of perceived exertion, work rate, heart rate, or oxygen uptake during exercise. Recovery of oxygen uptake and heart rate after the 30 min exercise followed a similar time course in the ginger and placebo conditions.	No
Maghbooli *et al.* (2014) [43]	Time of headache onset, severity, and response self-assessments	Ginger showed similar effect to sumatriptan when patients relieved migraine attack.	No
Arablou *et al.* (2013) [44]	Anthropometric indices[1], glycemic and lipid profile	There were significant differences between ginger intervention group and placebo group in serum FPG, HbA1c, Insulin, HOMA, TG, TC, CRP, PGE_2 while there were no significant differences in HDL, LDL and TNF-alpha.	No
Mozaffari-Khosravi *et al.* (2014) [45]	FBS, HbA1c, fructosamine, QUICKI, BMI, fasting insulin, HOMA-IR, insulin sensitivity, beta-cell function	FBS decreased 10.5% in ginger intake group while it increased 21% in placebo group. There is significant difference in fasting insulin level, insulin sensitivity, HOMA-IR, QUICKI more increased in ginger group significantly.	No
Andallu *et al.* (2001) [46]	Body weight, blood glucose, total cholesterol, triglycerides, HDL cholesterol, LDL cholesterol, VLDL cholesterol	Ginger treatment may be helpful in reducing blood glucose and serum lipid levels in diabetic or hypercholesterolemic people.	No
Karimi *et al.* (2013) [47]	Adiponectin, glutathione peroxidase, nitric oxide, MDA	It was concluded that ginger could reduce oxidative stress in obese women who were diagnosed with breast cancer.	No
Atashak *et al.* (2011) [48]	CRP, HOMA-IR, Insulin, blood lipids (total cholesterol, LDL-C, HDL-C, TG), anthropometric measurements	This study showed that treating ginger supplements alone or combination may reduce chronic inflammation.	No
Alizadeh-Navaei *et al.* (2008) [49]	Lipid levels (fasting serum TG, cholesterol, HDL, LDL, and VLDL), fasting lipoprotein-α, fasting homocysteine	The mean change level of triglyceride and cholesterol was significantly higher in ginger group than control group. In addition, the significant reduction of TC, cholesterol, LDL, VLDL, lipoprotein-α and homocysteine were observed when comparing the before and after ginger treatment.	No

Imani *et al.* (2014) [50]	Serum fasting glucose, carboxymethyl lysine, pentosidine, MDA, hs-CRP, sICAM-1, sVCAM-1, sE-selectin	Administrating 1000 mg ginger daily reduced serum fasting glucose in patients undergoing peritoneal dialysis.	No
Hu *et al.* (2011) [51]	Antral area, antral contractions, fundic area and diameter, and gastric half-emptying time (T50)	Gastric half-emptying time was more rapid in ginger treatment group than placebo group. There is no significant difference in any other parameters.	No
Wu *et al.* (2007) [52]	Antral area, antral contraction, gastric half-emptying time, fundic area and diameter, gastrointestinal sensations score (by VAS)	In ginger treatment group, the antral area and gastric half-emptying time were decreased more rapidly while the frequency of antral contractions was increased.	No
Lien *et al.* (2002) [52]	Vection-induced gastric dysrhythmias (EGG), vection-induced elevation of plasma vasopressin, vasopressin-evoked nausea, vasopressin-evoked gastric dysrhythmias	Ginger treatment has the effect in reducing the severity of nausea, tachygastric activity, and vasopressin release.	No
Shariatpanahi *et al.* (2009) [54]	Mean nutritional intake on first 48 h, Mean nutritional intake on total period of study, ICU free days, ventilator-free days, mortality, nosocomial pneumonia	Reducing delayed gastric emptying, eliminating the use of transpyloric feeding and preventing the development of nosocomial pneumonia can be achieved by gastric feeding with ginger extract.	No
Gonlachanvit *et al.* (2003) [55]	Plasma glucose level, electrogastrography, hyperglycemic clamping technique	Ginger root could prevent slow-wave dysrhythmias evoked by acute hyperglycemia. However, no influence was recorded on dysrhythmias induced by a prostaglandin E1 analog.	No
Haghighi *et al.* (2005) [56]	Severity of pain, gelling pain score, joint swelling score, joint motion slope score	Regarding relieving symptoms of osteoarthritis, the ginger and ibuprofen groups were significantly more affected than the placebo group, however, there was no significant difference between the ginger and ibuprofen groups.	No
Paramdeep *et al.* (2013) [57]	WOMAC index, pain (by VAS)	this study indicates that ginger added additional effect regarding analgesic and anti-inflammatory effect in patients of Osteoarthritis.	Yes
Drozdov *et al.* (2012) [58]	PGE1, PGE2, PGF2α, PGI2, serum gastrin-17 levels severity of dyspepsia assessment form, arthritic pain (by VAS)	Treatment combination of ginger and glucosamine was as effective as NSAID in OA patients and may have an additional gastro protective effect.	No
Bliddal *et al.* (2000) [59]	Pain assessment (by VAS), Lequesne-index for either hip or knee, range of motion	The result suggested that the treating ginger to deal with the pain and dysfunction of osteoarthritis of the hip or knee is more effective than placebo but less effective than ibuprofen.	No
Gregersen *et al.* (2013) [60]	Diet-induced thermogenesis, respiratory quotient, and substrate oxidation, catecholamines, hemodynamic factors, ad libitum energy intake, appetite measures, energy balance, blood parameters	At palatable levels in food, ginger had no significant effects on any of the primary or secondary outcome measures relating to energy metabolism, appetite or food intake.	No
Mansour *et al.* (2012) [61]	Increase of Thermic Effect of Food (difference between post-prandial energy expenditure and resting metabolic rate), AUC for Thermic Effect of Food, post-prandial energy expenditure, respiratory quotient,	Ginger may have a potential effect on weight management by enhancing thermogenesis and reducing feelings of hunger.	No

	AUC of VAS ratings of hunger, AUC for VAS ratings of food intake, AUC for VAS ratings of greater fullness, glucose, insulin, lipids, or inflammatory markers		
Janssen *et al.* (1996) [62]	Maximum ex vivo platelet thromboxane B2 production	It was concluded that ginger intake had no significant effect on anti-thrombotic.	No
Bordia, *et al.* (1996) [63]	Adenosine diphosphate-induced platelet aggregation, epinephrine-induced platelet aggregation, blood lipids, blood sugar	A 4 g daily of ginger intake showed no effect on adenosine diphosphate- and epinephrine-induced platelet aggregation, blood lipid, and blood sugar. 10 g of single dose after 4 h had a significant effect in reducing platelet aggregation.	No
Jiang *et al.* (2004) [64]	Pharmacokinetic parameters, pharmacodynamic parameters Platelet aggregation	Ginger did not show significant effect on the pharmacokinetics or pharmacodynamics of warfarin and clotting status.	No
Shariatpanahi *et al.* (2013) [65]	IL-1, IL-6, TNF-alpha, LTB4, red blood cell glutathione, organ failure-free days	Patients consumed ginger have a significant lower level of IL-1, IL-6, and TNF alpha but significant higher level of red blood cell glutathione. Ginger treatment group has less duration of mechanical ventilation and length of intensive care unit stay.	No
Rouhi *et al.* (2006) [66]	Dyspnea, wheezing, chest tightness, stage of disease, nocturnal cough, spray dosage, dyspnea attack, FEF25-75, FEV1, FVC	This study showed that 1-4 g of ginger is effective in reducing asthmatic symptoms, but ineffective in spirometry findings and changing the stage of the disease.	No

1: Body weight, BMI, serum FPG, HbA1c, Insulin, HOMA, TG, TC, HDL, LDL, CRP, TNF-alpha, PGE_2

CINV: chemotherapy-induced nausea and vomiting; CRP: C-reactive protein; FBS: fasting blood sugar; FLIE-5DR: functional living index emesis 5 day recall; hs-CRP: high-sensitivity C-reactive protein; HOMA-IR: homeostasis model assessment of insulin resistance; HDL-C: high density lipoprotein-cholesterol; HbA1c: Hemoglobin A1c; IL: interleukin; LDL-C: low density lipoprotein-cholesterol; MDA: malondialdehyde; PVAS: pain visual analog scale; QUICKI: quantitative insulin sensitivity check index; RINVR: Rhodes Inventory of Nausea, Vomiting and Retching; TNF-α: tumor necrosis factor alpha; TAC: total antioxidant capacity; VAS: visual analog scale; ESAS: edmonton's symptom assessment scale; PG: prostaglandins; sICAM-1: soluble intercellular adhesion molecule type 1; sVCAM-1: soluble vascular cell adhesion molecule type 1; WOMAC: Western Ontario and McMaster Universities osteoarthritis

1. Bossi, P.; Cortinovis, D.; Fatigoni, S.; Cossu Rocca, M.; Fabi, A.; Seminara, P.; Ripamonti, C.; Alfieri, S.; Granata, R.; Bergamini, C., et al. A randomized, double-blind, placebo-controlled, multicenter study of a ginger extract in the management of chemotherapy-induced nausea and vomiting (CINV) in patients receiving high-dose cisplatin. Annals of Oncology 2017, 28, 2547-2551, doi:10.1093/annonc/mdx315.
2. Konmun, J.; Danwilai, K.; Ngamphaiboon, N.; Sripanidkulchai, B.; Sookprasert, A.; Subongkot, S. A phase II randomized double-blind placebo-controlled study of 6-gingerol as an anti-emetic in solid tumor patients receiving moderately to highly emetogenic chemotherapy. Medical Oncology 2017, 34, 69, doi:10.1007/s12032-017-0931-4.
3. Zeraati, H.; Shahinfar, J.; Imani Hesari, S.; Masrorniya, M.; Nasimi, F. The Effect of Ginger Extract on the Incidence and Severity of Nausea and Vomiting After Cesarean Section Under Spinal Anesthesia. Anesth Pain Med 2016, 6, e38943-e38943, doi:10.5812/aapm.38943.
4. Adib Rad, H.; Basirat, Z.; Bakouei, F.; Moghadamnia, A.A.; Khafri, S.; Farhadi Kotenaei, Z.; Nikpour, M.; Kazemi, S. Effect of Ginger and Novafen on menstrual pain: A cross-over trial. Taiwanese Journal of Obstetrics and Gynecology 2018, 57, 806-809, doi:https://doi.org/10.1016/j.tjog.2018.10.006.
5. Shirvani, M.A.; Motahari-Tabari, N.; Alipour, A. Use of ginger versus stretching exercises for the treatment of primary dysmenorrhea: a randomized controlled trial. Journal of Integrative Medicine 2017, 15, 295-301, doi:https://doi.org/10.1016/S2095-4964(17)60348-0.
6. Mashak, B.; Hashemnejad, M.; Kabir, K.; Refaei, M.; Saeieh, S.; Torkashvand, S.; Salehi, L.; Yazdkhasti, M.J.I.J.o.W.s.H.; Sciences, R. The effect of ginger on preventing post-spinal puncture headache in patients undergoing cesarean section. 2019, 7, 204-210.
7. Hashemi, M.; Ghasemi, M.; Taheri, M.; Dadkhah, P.J.E.J.o.G.M. Comparing the effect of ginger and vitamin D3 supplement on inflammatory factors and pain severity in adults with low back pain. 2019, 16.
8. Hasanvand, A.; Ebrahimi, Y.; Mohamadi, A.; Nazari, A.J.J.o.H.P. Zingiber officinale Roscoe reduces chest pain on patients undergoing coronary angioplasty: a clinical trial. 2019, 8.
9. Azimi, P.; Ghiasvand, R.; Feizi, A.; Hosseinzadeh, J.; Bahreynian, M.; Hariri, M.; Khosravi-Boroujeni, H. Effect of cinnamon, cardamom, saffron and ginger consumption on blood pressure and a marker of endothelial function in patients with type 2 diabetes mellitus: A randomized controlled clinical trial. Blood Pressure 2016, 25, 133-140, doi:10.3109/08037051.2015.1111020.
10. Shidfar, F.; Rajab, A.; Rahideh, T.; Khandouzi, N.; Hosseini, S.; Shidfar, S.J.J.o.c.; medicine, i. The effect of ginger (Zingiber officinale) on glycemic markers in patients with type 2 diabetes. 2015, 12, 165-170.
11. Tabibi, H.; Imani, H.; Atabak, S.; Najafi, I.; Hedayati, M.; Rahmani, L.J.P.D.I. Effects of ginger on serum lipids and lipoproteins in peritoneal dialysis patients: a randomized controlled trial. 2016, 36, 140-145.
12. Karimi, N.; Dabidi Roshan, V.; Fathi Bayatiyani, Z. Individually and Combined Water-Based Exercise With Ginger Supplement, on Systemic Inflammation and Metabolic Syndrome Indices, Among the Obese Women With Breast Neoplasms. Iran J Cancer Prev 2015, 8, e3856-e3856, doi:10.17795/ijcp-3856.
13. Emrani, Z.; Shojaei, E.; Khalili, H. Ginger for Prevention of Antituberculosis-induced Gastrointestinal Adverse Reactions Including Hepatotoxicity: A Randomized Pilot Clinical Trial. Phytotherapy Research 2016, 30, 1003-1009, doi:10.1002/ptr.5607.
14. Kulkarni, R.A.; Deshpande, A.R.J.J.o.C.; Medicine, I. Anti-inflammatory and antioxidant effect of ginger in tuberculosis. 2016, 13, 201-206.
15. Miyamoto, M.; Matsuzaki, K.; Katakura, M.; Hara, T.; Tanabe, Y.; Shido, O. Oral intake of encapsulated dried ginger root powder hardly affects human thermoregulatory function, but appears to facilitate fat utilization. International Journal of Biometeorology 2015, 59, 1461-1474, doi:10.1007/s00484-015-0957-2.
16. Manusirivithaya, S.; Sripramote, M.; Tangjitgamol, S.; Sheanakul, C.; Leelahakorn, S.; Thavaramara, T.; Tangcharoenpanich, K. Antiemetic effect of ginger in gynecologic oncology patients receiving cisplatin. International Journal of Gynecologic Cancer 2004, 14, 1063, doi:10.1136/ijgc-00009577-200411000-00002.
17. Pillai, A.K.; Sharma, K.K.; Gupta, Y.K.; Bakhshi, S. Anti-emetic effect of ginger powder versus placebo as an add-on therapy in children and young adults receiving high emetogenic chemotherapy. Pediatric Blood & Cancer 2011, 56, 234-238, doi:10.1002/pbc.22778.
18. Panahi, Y.; Saadat, A.; Sahebkar, A.; Hashemian, F.; Taghikhani, M.; Abolhasani, E. Effect of Ginger on Acute and Delayed Chemotherapy-Induced Nausea and Vomiting: A Pilot, Randomized, Open-Label Clinical Trial. Integrative Cancer Therapies 2012, 11, 204-211, doi:10.1177/1534735411433201.
19. Sontakke, S.; Thawani, V.; Naik, M.J.I.j.o.p. Ginger as an antiemetic in nausea and vomiting induced by chemotherapy: a randomized, cross-over, double blind study. 2003, 35, 32-36.
20. Arslan, M.; Ozdemir, L.J.C.j.o.o.n. Oral intake of ginger for chemotherapy-induced nausea and vomiting among women with breast cancer. 2015, 19.

21. Unit, G.J.P.J.o.B.S. Treatment of Pregnancy Nausea. 2011, 14, 817-820.
22. Ozgoli, G.; Goli, M.; Simbar, M. Effects of Ginger Capsules on Pregnancy, Nausea, and Vomiting. The Journal of Alternative and Complementary Medicine 2009, 15, 243-246, doi:10.1089/acm.2008.0406.
23. Keating, A.; Chez, R.A.J.A.t.i.h.; medicine. Ginger syrup as an antiemetic in early pregnancy. 2002, 8, 89.
24. Pongrojpaw, D.; Somprasit, C.; Chanthasenanont, A.J.J.-M.A.o.T. A randomized comparison of ginger and dimenhydrinate in the treatment of nausea and vomiting in pregnancy. 2007, 90, 1703.
25. Saberi, F.; Sadat, Z.; Abedzadeh-Kalahroudi, M.; Taebi, M. Effect of ginger on relieving nausea and vomiting in pregnancy: a randomized, placebo-controlled trial. Nurs Midwifery Stud 2014, 3, e11841-e11841, doi:10.17795/nmsjournal11841.
26. Haji Seid Javadi, E.; Salehi, F.; Mashrabi, O.J.O.; international, g. Comparing the effectiveness of vitamin b6 and ginger in treatment of pregnancy-induced nausea and vomiting. 2013, 2013.
27. Apariman, S.; Ratchanon, S.; Wiriyasirivej, B.J.J.-M.A.o.T. Effectiveness of ginger for prevention of nausea and vomiting after gynecological laparoscopy. 2006, 89, 2003.
28. Nanthakomon, T.; Pongrojpaw, D.J.J.M.A.T. The efficacy of ginger in prevention of postoperative nausea and vomiting after major gynecologic surgery. 2006, 89, S130-136.
29. Kalava, A.; Darji, S.J.; Kalstein, A.; Yarmush, J.M.; SchianodiCola, J.; Weinberg, J. Efficacy of ginger on intraoperative and postoperative nausea and vomiting in elective cesarean section patients. European Journal of Obstetrics & Gynecology and Reproductive Biology 2013, 169, 184-188, doi:https://doi.org/10.1016/j.ejogrb.2013.02.014.
30. Visalyaputra, S.; Petchpaisit, N.; Somcharoen, K.; Choavaratana, R. The efficacy of ginger root in the prevention of postoperative nausea and vomiting after outpatient gynaecological laparoscopy. Anaesthesia 1998, 53, 506-510, doi:10.1046/j.1365-2044.1998.00369.x.
31. Phillips, S.; Ruggier, R.; Hutchinson, S.E. Zingiber officinale (Ginger)–an antiemetic for day case surgery. Anaesthesia 1993, 48, 715-717, doi:10.1111/j.1365-2044.1993.tb07188.x.
32. Bone, M.E.; Wilkinson, D.J.; Young, J.R.; McNeil, J.; Charlton, S. Ginger root—a new antiemetic The effect of ginger root on postoperative nausea and vomiting after major gynaecological surgery. Anaesthesia 1990, 45, 669-671, doi:10.1111/j.1365-2044.1990.tb14395.x.
33. Tavlan, A.; Tuncer, S.; Erol, A.; Reisli, R.; Aysolmaz, G.; Otelcioglu, S. Prevention of Postoperative Nausea and Vomiting after Thyroidectomy. Clinical Drug Investigation 2006, 26, 209-214, doi:10.2165/00044011-200626040-00005.
34. Dabaghzadeh, F.; Khalili, H.; Dashti-Khavidaki, S.; Abbasian, L.; Moeinifard, A. Ginger for prevention of antiretroviral-induced nausea and vomiting: a randomized clinical trial. Expert Opinion on Drug Safety 2014, 13, 859-866, doi:10.1517/14740338.2014.914170.
35. Holtmann, S.; Clarke, A.H.; Scherer, H.; Höhn, M. The Anti-motion Sickness Mechanism of Ginger: A Comparative Study with Placebo and Dimenhydrinate. Acta Oto-Laryngologica 1989, 108, 168-174, doi:10.3109/00016488909125515.
36. Grøntved, A.; Brask, T.; Kambskard, J.; Hentzer, E. Ginger Root Against Seasickness: A Conctrolled Trial on the Open Sea. Acta Oto-Laryngologica 1988, 105, 45-49, doi:10.3109/00016488809119444.
37. Grøntved, A.; Hentzer, E. Vertigo-Reducing Effect of Ginger Root. ORL 1986, 48, 282-286, doi:10.1159/000275883.
38. Mowrey, D.; Clayson, D. MOTION SICKNESS, GINGER, AND PSYCHOPHYSICS. The Lancet 1982, 319, 655-657, doi:https://doi.org/10.1016/S0140-6736(82)92205-X.
39. Stewart, J.J.; Wood, M.J.; Wood, C.D.; Mims, M.E. Effects of Ginger on Motion Sickness Susceptibility and Gastric Function. Pharmacology 1991, 42, 111-120, doi:10.1159/000138781.
40. Jenabi, E.J.J.P.M.A. The effect of ginger for relieving of primary dysmenorrhoea. 2013, 63, 8-10.
41. Shirvani, M.A.; Motahari-Tabari, N.; Alipour, A. The effect of mefenamic acid and ginger on pain relief in primary dysmenorrhea: a randomized clinical trial. Archives of Gynecology and Obstetrics 2015, 291, 1277-1281, doi:10.1007/s00404-014-3548-2.
42. Christopher, D.B.; Patrick, J.O.C. Acute Effects of Dietary Ginger on Quadriceps Muscle Pain during Moderate-Intensity Cycling Exercise. International Journal of Sport Nutrition and Exercise Metabolism 2008, 18, 653-664, doi:10.1123/ijsnem.18.6.653.
43. Maghbooli, M.; Golipour, F.; Moghimi Esfandabadi, A.; Yousefi, M. Comparison Between the Efficacy of Ginger and Sumatriptan in the Ablative Treatment of the Common Migraine. Phytotherapy Research 2014, 28, 412-415, doi:10.1002/ptr.4996.
44. Arablou, T.; Aryaeian, N.; Valizadeh, M.; Sharifi, F.; Hosseini, A.; Djalali, M. The effect of ginger consumption on glycemic status, lipid profile and some inflammatory markers in patients with type 2 diabetes

mellitus. International Journal of Food Sciences and Nutrition 2014, 65, 515-520, doi:10.3109/09637486.2014.880671.
45. Mozaffari-Khosravi, H.; Talaei, B.; Jalali, B.-A.; Najarzadeh, A.; Mozayan, M.R. The effect of ginger powder supplementation on insulin resistance and glycemic indices in patients with type 2 diabetes: A randomized, double-blind, placebo-controlled trial. Complementary Therapies in Medicine 2014, 22, 9-16, doi:https://doi.org/10.1016/j.ctim.2013.12.017.
46. Andallu, B.; Radhika, B.; Suryakantham, V. Effect of aswagandha, ginger and mulberry on hyperglycemia and hyperlipidemia. Plant Foods for Human Nutrition 2003, 58, 1-7, doi:10.1023/B:QUAL.0000040352.23559.04.
47. Karimi, N.; Roshan, V.D.J.A.P.J.C.P. Change in adiponectin and oxidative stress after modifiable lifestyle interventions in breast cancer cases. 2013, 14, 2845-2850.
48. Atashak, S.; Peeri, M.; Azarbayjani, M.A.; Stannard, S.R.; Haghighi, M.M. Obesity-related cardiovascular risk factors after long- term resistance training and ginger supplementation. J Sports Sci Med 2011, 10, 685-691.
49. Alizadeh-Navaei, R.; Roozbeh, F.; Saravi, M.; Pouramir, M.; Jalali, F.; Moghadamnia, A.A.J.S.M.J. Investigation of the effect of ginger on the lipid levels. A double blind controlled clinical trial. 2008, 29, 1280-1284.
50. Imani, H.; Tabibi, H.; Najafi, I.; Atabak, S.; Hedayati, M.; Rahmani, L. Effects of ginger on serum glucose, advanced glycation end products, and inflammation in peritoneal dialysis patients. Nutrition 2015, 31, 703-707, doi:https://doi.org/10.1016/j.nut.2014.11.020.
51. Hu, M.-L.; Rayner, C.K.; Wu, K.-L.; Chuah, S.-K.; Tai, W.-C.; Chou, Y.-P.; Chiu, Y.-C.; Chiu, K.-W.; Hu, T.-H. Effect of ginger on gastric motility and symptoms of functional dyspepsia. World J Gastroenterol 2011, 17, 105-110, doi:10.3748/wjg.v17.i1.105.
52. Wu, K.-L.; Rayner, C.K.; Chuah, S.-K.; Changchien, C.-S.; Lu, S.-N.; Chiu, Y.-C.; Chiu, K.-W.; Lee, C.-M.J.E.j.o.g.; hepatology. Effects of ginger on gastric emptying and motility in healthy humans. 2008, 20, 436-440.
53. Lien, H.-C.; Sun, W.M.; Chen, Y.-H.; Kim, H.; Hasler, W.; Owyang, C.J.A.J.o.P.-G.; Physiology, L. Effects of ginger on motion sickness and gastric slow-wave dysrhythmias induced by circular vection. 2003, 284, G481-G489.
54. Shariatpanahi, Z.V.; Taleban, F.A.; Mokhtari, M.; Shahbazi, S. Ginger extract reduces delayed gastric emptying and nosocomial pneumonia in adult respiratory distress syndrome patients hospitalized in an intensive care unit. Journal of Critical Care 2010, 25, 647-650, doi:https://doi.org/10.1016/j.jcrc.2009.12.008.
55. Gonlachanvit, S.; Chen, Y.H.; Hasler, W.L.; Sun, W.M.; Owyang, C. Ginger Reduces Hyperglycemia-Evoked Gastric Dysrhythmias in Healthy Humans: Possible Role of Endogenous Prostaglandins. Journal of Pharmacology and Experimental Therapeutics 2003, 307, 1098, doi:10.1124/jpet.103.053421.
56. Haghighi, M.; Khalvat, A.; Toliat, T.; Jallaei, S. Comparing the effects of ginger (Zingiber officinale) extract and ibuprofen on patients with osteoarthritis. 2005.
57. Paramdeep, G.J.I.J.P.P. Efficacy and tolerability of ginger (Zingiber officinale) in patients of osteoarthritis of knee. 2013, 57, 177-183.
58. Drozdov, V.N.; Kim, V.A.; Tkachenko, E.V.; Varvanina, G.G. Influence of a Specific Ginger Combination on Gastropathy Conditions in Patients with Osteoarthritis of the Knee or Hip. The Journal of Alternative and Complementary Medicine 2012, 18, 583-588, doi:10.1089/acm.2011.0202.
59. Bliddal, H.; Rosetzsky, A.; Schlichting, P.; Weidner, M.S.; Andersen, L.A.; Ibfelt, H.H.; Christensen, K.; Jensen, O.N.; Barslev, J. A randomized, placebo-controlled, cross-over study of ginger extracts and Ibuprofen in osteoarthritis. Osteoarthritis and Cartilage 2000, 8, 9-12, doi:https://doi.org/10.1053/joca.1999.0264.
60. Gregersen, N.T.; Belza, A.; Jensen, M.G.; Ritz, C.; Bitz, C.; Hels, O.; Frandsen, E.; Mela, D.J.; Astrup, A. Acute effects of mustard, horseradish, black pepper and ginger on energy expenditure, appetite, ad libitum energy intake and energy balance in human subjects. British Journal of Nutrition 2013, 109, 556-563, doi:10.1017/S0007114512001201.
61. Mansour, M.S.; Ni, Y.-M.; Roberts, A.L.; Kelleman, M.; RoyChoudhury, A.; St-Onge, M.-P. Ginger consumption enhances the thermic effect of food and promotes feelings of satiety without affecting metabolic and hormonal parameters in overweight men: A pilot study. Metabolism 2012, 61, 1347-1352, doi:https://doi.org/10.1016/j.metabol.2012.03.016.
62. Janssen, P.; Meyboom, S.; Van Staveren, W.; De Vegt, F.; Katan, M.J.E.J.o.C.N. Consumption of ginger (Zingiber officinale roscoe) does not affect ex vivo platelet thromboxane production in humans. 1996, 50, 772-774.
63. Bordia, A.; Verma, S.K.; Srivastava, K.C. Effect of ginger (Zingiber officinale Rosc.) and fenugreek (Trigonella foenumgraecum L.) on blood lipids, blood sugar and platelet aggregation in patients with coronary artery disease. Prostaglandins, Leukotrienes and Essential Fatty Acids 1997, 56, 379-384, doi:https://doi.org/10.1016/S0952-3278(97)90587-1.
64. Jiang, X.; Williams, K.M.; Liauw, W.S.; Ammit, A.J.; Roufogalis, B.D.; Duke, C.C.; Day, R.O.; McLachlan,

A.J. Effect of ginkgo and ginger on the pharmacokinetics and pharmacodynamics of warfarin in healthy subjects. British Journal of Clinical Pharmacology 2005, 59, 425-432, doi:10.1111/j.1365-2125.2005.02322.x.
65. Vahdat Shariatpanahi, Z.; Mokhtari, M.; Taleban, F.A.; Alavi, F.; Salehi Surmaghi, M.H.; Mehrabi, Y.; Shahbazi, S. Effect of enteral feeding with ginger extract in acute respiratory distress syndrome. Journal of Critical Care 2013, 28, 217.e211-217.e216, doi:https://doi.org/10.1016/j.jcrc.2012.04.017.
66. Rouhi, H.; Ganji, F.; Nasri, H.J.P.J.N. Effects of Ginger on the improvement of asthma [the evaluation of Its treatmental effects]. 2006, 5, 373-376.

Table S4. Types of adverse effect, incidence rate, and dosage from 17 trials.

Reference	Reported adverse effect	Reported case	Dosage (per day)
**Kashefi *et al.* (2013)**	Headache	2.17%	750 mg
	Diarrhea	0%	
	Heartburn	3.26%	
**Kashefi *et al.* (2015)**	Heartburn	2.17%	750 mg
	Abdominal pain	2.17%	
	Diarrhea	2.17%	
**Aryaeian *et al.* (2019)**	Heartburn	3.03%	1500 mg
**Mahluji *et al.* (2013)**	Heartburn	3.57%	2000 mg
**Citronberg *et al.* (2013)**	Bloating	Overall, 70%	2000 mg
	Gas		
	Nausea		
	Heartburn		
**Wigler *et al.* (2003)**	Heartburn	6.90%	1000 mg
**Paramdeep *et al.* (2013)**	Nausea	10.00%	750 mg
	Epigastric distress	10.00%	
	Heartburn	10.00%	
**Kalava *et al.* (2013)**	Heartburn	7.00%	1000 mg
	Diarrhea	4.00%	
	Mouth irritation	1.00%	
**Ryan *et al.* (2012)**	Heartburn	N/A	250 mg, 500 mg or 750 mg
	Bruising/flushing	N/A	
	Rash	N/A	
**Eberhart *et al.* (2003)**	Flu-like symptom	1.69% (300 mg/d), 1.75% (600 mg/d)	300 mg or 600 mg
	Heartburn	1.69% (300 mg/d), 3.51% (600 mg/d)	
	Cardiovascular symptom	0% (300 mg/d), 3.51% (600 mg/d)	
	Respiratory symptom	3.39% (300 mg/d), 0% (600 mg/d)	

	Infection	5.08% (300 mg/d), 0% (600 mg/d)	
**Emrani *et al.* (2016)**	Heartburn	10.00%	500 mg
**Tilburg *et al.* (2014)**	Not sure about the ginger's side effect[1]	N/A	1000 mg or 2000 mg
**Zick *et al.* (2014)**	Not sure about the ginger's side effect[2]	N/A	2000 mg
**Pongrojpaw *et al.* (2007)**	Drowsiness	5.88%	1000 mg
	Heartburn	15.29%	
**Firouzbakht *et al.* (2014)**	Abdominal pain	10.20%	1000 mg
	Nausea		
	Heartburn		
**Stewart *et al.* (1990)**	Gastric burning	37.50%	500 mg or 1000 mg
	Intense urge to urinate	25.00%	
**Yekta *et al.* (2012)**	Heartburn	10.00% (anticipatory), 12.50% (acute), 5.00% (delayed)	1000 mg

1: Headache, tiredness, heartburn, nausea, difficulty passing stool, more frequent stools, loose stools, bloating and hunger suppression
2: Bloating, urgency, gas, nausea, heartburn, sores in mouth and anorexia

Table S5. Quality assessment outcomes and quality assessment scores of the included studies.

Author (year)	Random sequence generation	Allocation concealment	Blinding of participants and personnel*	Blinding of outcome assessment*	Incomplete outcome data*	Selective reporting	Other bias	QA score (L=2, U=1, H=0)
**Black *et al.* (2010)**	L	L	L	H	L	L	L	12
**Black *et al.* (2008)**	U	L	L	H	L	L	H	9
**Gregersen *et al.* (2013)**	L	H	U	L	L	L	H	9
**Manusirivithaya *et al.* (2004)**	U	H	L	H	L	L	H	7
**Pillai *et al.* (2011)**	L	H	L	H	L	L	U	9
**Kulkarni *et al.* (2016)**	U	H	U	H	L	L	H	6
**Holtmann *et al.* (1989)**	U	H	L	H	L	L	H	7
**Karimi *et al.* (2013)**	U	H	U	H	L	L	H	6
**Attari,*et al.* (2016)**	U	L	L	H	L	L	L	11
**Ensiyeh *et al.* (2009)**	L	L	L	H	L	L	L	12
**Hashemi *et al.* (2019)**	U	L	L	H	U	L	H	8

**Haghighi** ***et al.*** **(2005)**	U	H	L	H	L	L	H	7
**Mohammadbeigi** ***et al.*** **(2011)**	L	H	L	H	L	L	H	8
**Sharifzadeh** ***et al.*** **(2018)**	L	L	L	L	L	L	H	12
**Maghbooli** ***et al.*** **(2014)**	U	L	L	H	L	L	H	9
**Ozgoli** ***et al.*** **(2009)**	H	L	L	L	L	L	L	12
**Kashefi** ***et al.*** **(2013)**	L	L	L	L	H	H	L	10
**Janssen** ***et al.*** **(1996)**	U	L	U	H	L	L	H	8
**Martins** ***et al.*** **(2018)**	L	H	L	L	L	L	L	12
**Arfeen** ***et al.*** **(1995)**	U	L	L	L	L	L	H	11
**Willetts** ***et al.*** **(2003)**	L	L	L	L	L	L	H	12
**Marx** ***et al.*** **(2017)**	L	H	L	L	L	L	L	12
**Azimi** ***et al.*** **(2016)**	U	H	L	H	L	L	L	9
**Shariatpanahi** ***et al.*** **(2013)**	U	H	L	L	L	L	H	9

**Bordia *et al.* (1997)**	U	H	U	H	L	L	H	6
**Shidfar *et al.* (2015)**	U	U	L	U	L	L	H	9
**Kashefi *et al.* (2015)**	L	L	L	L	L	L	L	14
**Sanaati *et al.* (2016)**	L	L	L	L	L	L	L	14
**Rad *et al.* (2018)**	U	H	L	H	L	L	L	9
**Arablou *et al.* (2013)**	U	H	L	H	L	L	H	7
**Jenabi (2013)**	L	L	U	H	H	L	H	7
**Panahi *et al.* (2012)**	H	H	H	H	L	H	L	4
**Paritakul *et al.* (2016)**	L	L	L	L	L	L	L	14
**Hu *et al.* (2011)**	U	H	L	H	L	L	L	9
**Mashak *et al.* (2018)**	U	L	H	H	L	L	L	9
**Mozaffari-Khosravi *et al.* (2014)**	L	H	L	H	L	H	L	8
**Jiang *et al.* (2013)**	L	L	L	L	L	L	L	14

**Mozaffari-Khosravi *et al.* (2016)**	L	H	L	L	L	L	L	12
**Aryaeian *et al.* (2019)**	L	L	L	L	L	L	U	13
**Jiang *et al.* (2004)**	U	H	H	H	L	L	H	5
**Shirvani *et al.* (2014)**	U	H	U	H	L	H	L	6
**Rahnama *et al.* (2012)**	L	L	L	L	L	L	L	14
**Attari *et al.* (2015)**	L	L	L	H	L	L	L	12
**Apariman *et al.* (2006)**	L	H	U	H	L	L	L	9
**Mahluji *et al.* (2013)**	U	L	L	L	L	L	U	12
**Ozgoli *et al.* (2009)**	U	H	L	H	L	L	L	9
**Wu *et al.* (2007)**	U	H	L	H	L	L	U	8
**Lien *et al.* (2002)**	U	H	L	H	L	L	H	7
**Imani *et al.* (2014)**	L	H	L	H	L	L	H	8
**Tabibi *et al.* (2015)**	L	H	L	H	L	L	H	8
**Rouhi *et al.* (2006)**	U	H	U	H	L	L	H	6

**Citronberg *et al.* (2013)**	L	H	L	L	L	L	L	12
**Matsumura *et al.* (2015)**	L	H	L	U	U	L	L	10
**Wigler *et al.* (2003)**	L	L	L	L	L	L	L	14
**Paramdeep (2013)**	U	H	H	H	L	L	H	5
**Thamlikitkul *et al.* (2016)**	L	H	L	L	L	L	U	11
**Li *et al.* (2017)**	L	L	L	L	L	L	L	14
**Ansari *et al.* (2016)**	U	U	L	L	L	L	H	10
**Nanthakomon *et al.* (2006)**	U	U	L	H	L	L	H	8
**Kalava *et al.* (2013)**	U	H	L	H	L	L	L	9
**Visalyaputra *et al.* (1997)**	U	H	L	L	L	L	H	9
**Phillips *et al.* (1993)**	U	H	L	H	L	L	H	7
**Ryan *et al.* (2012)**	L	H	L	H	L	L	L	10
**Black *et al.* (2009)**	L	H	L	L	L	L	H	10

**Sontakke** ***et al.*** **(2002)**	U	H	L	H	L	L	H	7
**Mansour** ***et al.*** **(2012)**	U	H	H	H	L	L	L	7
**Eberhart** ***et al.*** **(2003)**	L	L	L	L	L	L	H	12
**Shariatpanahi** ***et al.*** **(2009)**	U	H	L	H	L	L	H	7
**Vutyavanich** ***et al.*** **(2001)**	U	L	L	H	L	L	U	10
**Dabaghzadeh** ***et al.*** **(2014)**	U	H	L	H	L	L	L	9
**Emrani** ***et al.*** **(2016)**	U	H	L	H	L	L	L	9
**Bossi** ***et al.*** **(2017)**	L	H	L	H	L	H	H	6
**Gonlachanvit** ***et al.*** **(2003)**	U	H	L	H	L	L	H	7
**Grontved** ***et al.*** **(1988)**	U	L	L	H	L	L	H	9
**Bone** ***et al.*** **(1990)**	U	H	L	H	L	H	H	5
**Keating** ***et al.*** **(2002)**	L	H	L	H	L	L	U	9

**Fischer-Rasmussen *et al.* (1990)**	U	L	L	L	L	L	H	11
**Drozdov *et al.* (2012)**	L	H	U	H	L	L	H	7
**Alizadeh-Navaei *et al.* (2008)**	U	H	L	H	L	L	U	8
**Tilburg *et al.* (2014)**	L	L	L	H	L	L	L	12
**Atashak *et al.* (2011)**	U	H	L	H	L	L	H	7
**Miyamoto *et al.* (2015)**	U	H	U	H	L	L	H	6
**Arslan *et al.* (2014)**	L	H	H	H	L	L	H	6
**Konmun *et al.* (2017)**	U	H	L	H	L	L	H	7
**Zick *et al.* (2011)**	L	L	L	L	L	L	H	12
**Zick *et al.* (2008)**	L	L	L	L	L	L	L	14
**Suzanna *et al.* (2014)**	L	L	L	H	L	L	L	12
**Tavlan *et al.* (2006)**	L	U	L	H	L	L	H	9

**Pongrojpaw** ***et al.*** **(2007)**	U	H	L	H	L	L	H	7
**Smith** ***et al.*** **(2004)**	L	U	L	L	L	L	H	11
**Bliddal** ***et al.*** **(2000)**	U	H	L	L	L	L	H	9
**Shirvani** ***et al.*** **(2017)**	L	H	U	H	L	L	L	9
**Grontved** ***et al.*** **(1985)**	U	H	L	H	U	L	H	6
**Phillips** ***et al.*** **(1992)**	L	L	L	H	L	L	H	10
**Hasanvand** ***et al.*** **(2018)**	U	H	L	H	L	L	H	7
**Biswas** ***et al.*** **(2011)**	L	L	L	H	L	L	L	12
**Saberi** ***et al.*** **(2009)**	L	H	U	H	L	L	U	8
**Andallu** ***et al.*** **(2001)**	H	H	U	H	U	L	H	4
**Karimi** ***et al.*** **(2015)**	U	H	U	H	L	L	H	6
**Firouzbakht** ***et al.*** **(2014)**	L	L	L	H	L	L	H	10
**Javadi** ***et al.*** **(2013)**	U	H	U	H	H	L	H	4

**Fahimi** ***et al.*** **(2010)**	U	U	L	H	L	L	L	10
**Mandal** ***et al.*** **(2014)**	L	U	L	H	L	L	L	11
**Zeraati** ***et al.*** **(2016)**	U	H	L	H	L	L	H	7
**Mowrey** ***et al.*** **(1982)**	U	U	U	H	L	L	H	7
**Stewart** ***et al.*** **(1990)**	L	H	U	H	H	L	H	5
**Yekta** ***et al.*** **(2012)**	L	U	L	L	L	L	L	13
**Arzati** ***et al.*** **(2017)**	U	U	L	H	L	L	L	10
**Khandouzi** ***et al.*** **(2013)**	L	L	L	H	L	L	L	12

H: High risk of bias; L: Low risk of bias; U: Unclear risk of bias
